# Development trends of human organoid‐based COVID‐19 research based on bibliometric analysis

**DOI:** 10.1111/cpr.13496

**Published:** 2023-05-22

**Authors:** Minghui Li, Yuhan Yuan, Ting Zou, Zongkun Hou, Liang Jin, Bochu Wang

**Affiliations:** ^1^ Key Laboratory of Biorheological Science and Technology, Ministry of Education, College of Bioengineering Chongqing University Chongqing China; ^2^ Southwest Hospital/Southwest Eye Hospital Third Military Medical University (Army Medical University) Chongqing China; ^3^ School of Basic Medical Sciences/School of Biology and Engineering (School of Modern Industry for Health and Medicine) Guizhou Medical University Guiyang China

## Abstract

Coronavirus disease 2019 (COVID‐19), a global pandemic caused by the severe acute respiratory syndrome coronavirus 2 (SARS‐CoV‐2), has posed a catastrophic threat to human health worldwide. Human stem cell‐derived organoids serve as a promising platform for exploring SARS‐CoV‐2 infection. Several review articles have summarized the application of human organoids in COVID‐19, but the research status and development trend of this field have seldom been systematically and comprehensively studied. In this review, we use bibliometric analysis method to identify the characteristics of organoid‐based COVID‐19 research. First, an annual trend of publications and citations, the most contributing countries or regions and organizations, co‐citation analysis of references and sources and research hotspots are determined. Next, systematical summaries of organoid applications in investigating the pathology of SARS‐CoV‐2 infection, vaccine development and drug discovery, are provided. Lastly, the current challenges and future considerations of this field are discussed. The present study will provide an objective angle to identify the current trend and give novel insights for directing the future development of human organoid applications in SARS‐CoV‐2 infection.

## Graphical abstract

Keyword co‐occurrence overlay visualization of the research of organoids for SARS‐CoV‐2 infection. More studies focused on pulmonary infection using organoid models at the early stages of the COVID‐19 pandemic. Recently, keywords included ‘drug repurposing’, ‘personalized medicine’, ‘repair’, ‘therapy’, ‘resistance’ and ‘therapeutics’ are more popular. Node size indicates the occurrence frequency; Node colour represents the keyword co‐occurrence based on their average publication year, purple means that keywords occurred earlier and marked with yellow stands that keywords are vigorous recently.

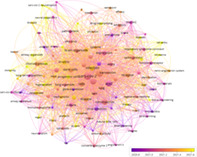

## 1 INTRODUCTION

The coronavirus disease 2019 (COVID‐19) pandemic, caused by the severe acute respiratory syndrome coronavirus 2 (SARS‐CoV‐2) infection, has posed a catastrophic threat to human health worldwide. Since its widely spread, as of 30 November 2022, roughly 639.1 million global cases were confirmed and 6.6 million deaths from COVID‐19.^1^ COVID‐19 cases could be asymptomatic or symptomatic, including fever or chills, cough, fatigue, shortness of breath and a loss of taste or sense of smell. Respiratory injury is the most common outcome in COVID‐19 patients, but SARS‐CoV‐2 can cause widespread damage to multi‐organs, including the eye, brain, heart, kidney, liver, lung and gastrointestinal tracts.^2^ To date, multiple SARS‐CoV‐2 variants have been described. For instance, the SARS‐CoV‐2 Omicron variant (B.1.1.529) was initially reported in South Africa and Botswana in November 2021.^3^, ^4^ Compared with the original coronavirus, the Omicron variant exhibits enhanced infectivity and transmissibility, high immune evasion ability and reduced pathogenicity.^3^, ^5^, ^6^ Although several COVID‐19 vaccines can prevent viral infections, new coronavirus variant may escape from immunity induced by the existing vaccines, leading to a great number of breakthrough infections.^7^, ^8^, ^9^ In this case, more efforts are needed to comprehensively decipher the pathophysiological processes of coronavirus infection and evaluate vaccine efficacy and safety.

Most current data on SARS‐CoV‐2 infection are limited to clinical data, classic cell experiments and restricted animal models.^10^, ^11^ Given angiotensin‐converting enzyme 2 (ACE2) functions as the key SARS‐CoV‐2 receptor, transgenic animals expressing human ACE2 are good options. However, animal‐based studies fail to faithfully mimic human physiology and recapitulate the human response to SARS‐CoV‐2 infection because of species variants. In addition, limited data reported how genetic diversity will influence the responses to SARS‐CoV‐2.^12^, ^13^ Human cell lines, including Caco‐2, Calu‐3, HEK293T, Huh7 and A549, have been utilized in viral infection experiments, whereas they are unable to adequately recapitulate the in vivo infection processes because monolayer cells lack cellular microenvironment, including cell–cell and cell–matrix interactions. Moreover, cancer cell lines carry the potential for malignant proliferation and uncontrollable mutations. Therefore, building human physiologically relevant model systems is dramatically demanded in the studies of viral infections.

Advanced stem cell technology has provided promising models in vitro to recapitulate human sophisticated, multicellular and physiological organs, namely organoids. They are three‐dimensional (3D) self‐organized tissues from human adult stem cells (ASCs) and pluripotent stem cells (PSCs), including human embryonic stem cells (hESCs) and induced pluripotent stem cells (hiPSCs). Human adult stem cell‐ or progenitor cell‐derived organoids could retain their organ identity, such as containing various cell types present in the native organ, recapitulating the cellular architecture and spatial organization of the organ and maintaining genetic stability over a long period of time.^14^ In contrast to the complicated and variable process of PSC differentiation, ASCs can be generated from biopsies isolated directly from healthy or patient tissue, thus providing a platform for personalized diagnosis and therapy.^15^, ^16^ ASC‐derived organoids are mainly generated from epithelial tissues (intestine, colon, stomach, liver, pancreas, lung, bladder and so forth) and need to isolate the tissue‐specific stem cell population, thus recapitulating only the epithelium of organs.^14^, ^15^, ^17^ ASC‐derived organoids are typically cystic and highly polarized epithelium, lacking nerves, blood vessels and stromal elements.^18^, ^19^, ^20^ Compared with ASC‐derived organoids, PSC‐derived organoids present higher complexity in structure.^20^ PSC‐derived organoids can be established from non‐epithelial tissues, including blood vessels, the brain and the retina.^21^, ^22^, ^23^ PSC‐derived embryoid bodies (EBs) can be guided to differentiate into three primary germ layers (ectoderm, mesoderm and endoderm), which recapitulates early embryonic development and finally generate a large variety of organoids.^15^, ^24^

Currently, various human organoids have been successfully established, such as brain, retinal, liver, kidney, stomach, intestinal, lung, cardiac and colon organoids and are widely applied for regenerative medicine and modelling human diseases, including infectious diseases. For instance, human brain organoid was utilized to understand Zika virus (ZIKV)‐induced adverse effects on the human brain. Qian's lab first generated brain organoids to elucidate ZIKV‐mediated neurodevelopmental disorder and model microcephaly.^25^ ZIKV was found to hijack host cells to increase viral replication and preferentially target neural progenitor cells, induce cell death, proliferation and premature differentiation of neural progenitors in human brain organoids.^25^, ^26^, ^27^ Furthermore, brain organoids were used for drug discovery against ZIKV infection.^28^ The study from Xu's group showed that Emricasan (a pan‐caspase inhibitor) and Niclosamide (The United States Food and Drug Administration (FDA)‐approved anthelmintic drug) were found to reduce ZIKV replication and protect neural progenitor cells from ZIKV‐induced cell death in brain organoids.^29^ Similarly, cholesterol‐25‐hydroxylase (CH25H) was observed to suppress viral infection and reduce tissue injuries in human ZIKV‐infected cortical organoids.^30^ These data stimulated the field to apply human organoids for studying viral infectivity, vaccine development and drug discovery of SARS‐CoV‐2.

**FIGURE 1:**
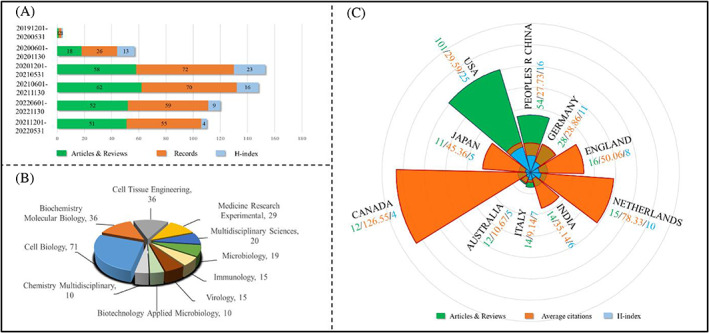
Characteristics of publications regarding human organoids applied in the COVID‐19 study. (A) Semi‐annual publications of human organoid application for SARS‐CoV‐2 infection. (B) Major fields of organoid application in COVID‐19 research. (C) The top 10 contributing countries/regions in organoid‐based COVID‐19 study.

Over the last 3 years, growing worldwide concerns regarding the application of human organoids for SARS‐CoV‐2 infection during the COVID‐19 epidemic scenario. Data from the Web of Science (WOS) Core Collection showed that the publication numbers dramatically increased, especially from 2020 to 2021 (Figure 1A). The major fields of organoid‐based COVID‐19 study are involved in Cell Biology, Biochemistry Molecular Biology, Cell Tissue Engineering, Medicine Research Experimental, Multidisciplinary Sciences, Microbiology, Immunology, Virology and so on (Figure 1B). Given the rapid development of organoid applications in SARS‐CoV‐2 infection, the research status and development trend of this field are systematically and comprehensively reviewed in the present study. Bibliometric analysis could be applied to determine the development, hotspots and trend directions of organoid applications in SARS‐CoV‐2 infection.^31^, ^32^, ^33^, ^34^ In this review, bibliometric method was used to reveal the research trends regarding the most influential countries and institutes, contributing journals and hotspots of the research field in organoid‐based COVID‐19 studies. We further provide a detail discussion on the application of human organoids in studying viral infectivity, vaccine development and drug discovery of SARS‐CoV‐2. At last, existing challenges and future perspectives in this field will be discussed. We hope this work will provide an objective angle to identify the current trend and give novel insights for guiding the future development of human organoid applications during the COVID‐19 pandemic scenario.

## 2 BIBLIOMETRIC ANALYSIS

### 2.1 Data source and search strategy

The WOS Core Collection Advanced Search based on Science Citation Index Expanded (SCI‐EXPANDED) and Social Sciences Citation Index (SSCI) was applied to retrieve records on organoid applications in SARS‐CoV‐2 research. The data was obtained from the period between 1 December 2019 and 27 November 2022, due to the first case occurred in Wuhan, China on 1 December 2019. Subsequently, WOS analysis and VOSviewer (version 1.6.18, from the Centre of Science and Technology Studies at Leiden University) application were applied to analyse the general information, including growing trends of publication and citation, the most productive/influential countries/regions and organizations, journal distribution, co‐cited references and sources and key research areas. The retrieval strategy was as follows: TS = (‘COVID‐19’ OR ‘coronavirus disease 2019’ OR ‘2019‐nCov’ OR ‘2019 novel coronavirus’ OR ‘SARS‐CoV‐2’ OR ‘Severe acute respiratory syndrome coronavirus 2’ OR ‘novel coronavirus disease 19’ OR ‘novel coronavirus disease‐19’ OR ‘SARS2’ OR ‘SARS‐2’ OR ‘COVID‐2019’ OR ‘COVID19’) AND (‘organoid*’). The inclusion criteria were publications written in English.

### 2.2 Publication and citation characteristics

A total of 284 records were retrieved, which included 154 original articles, 90 reviews, 18 editorials, 10 early access, 10 letters and 2 others. The articles and reviews constituted the largest share (54.22% and 31.69%, respectively) of the full share of the publications and were analysed in our subsequent analysis. As seen in Figure 1A, both the publications and citations of papers regarding organoid‐based COVID‐19 have increased rapidly over the last 3 years. This data suggests a growing interest in organoid applications in COVID‐19 research.

### 2.3 The most contributing countries/regions and organizations

Up to 45 countries or regions with publications related to organoid applications for SARS‐CoV‐2 infection. Figure 1C presents the top 10 contributing countries or regions in this field. As indicated, the United States ranks first with 101 publications and has the highest *H*‐index (25), which was normally utilized to evaluate the publication number and citation performance.^35^, ^36^ China (54 articles, 16) and Germany (28 articles, 11) rank second and third place, respectively. England, the Netherlands and India also are influential countries/regions in this field. Interestingly, in terms of average citations (citations per publication), Canada ranks first with 126.55 average times. The Netherlands (78.33) and England (50.06) rank second and third place, respectively. Regarding the most contributing organizations, 667 institutes around the world were identified associated with organoid‐based COVID‐19 study. As shown in Table S1, the University of California System and the Chinese Academy of Science rank first and second in terms of publication numbers, with 21 and 15 publications, respectively. The *H*‐indexes of the University of California System and the University of California San Diego are also in the leading place, reaching 12 and 9, respectively. Although the publications from the Karolinska Institutet put it in ninth place, it reached the highest average citations of 165.22. These top 10 contributing organizations are mainly from the United States, China and European countries. Taken together, scientists from the United States, China and European countries/regions paid great efforts in the application of organoids for SARS‐CoV‐2 infection.

### 2.4 Co‐citation analysis of references and sources

**FIGURE 2:**
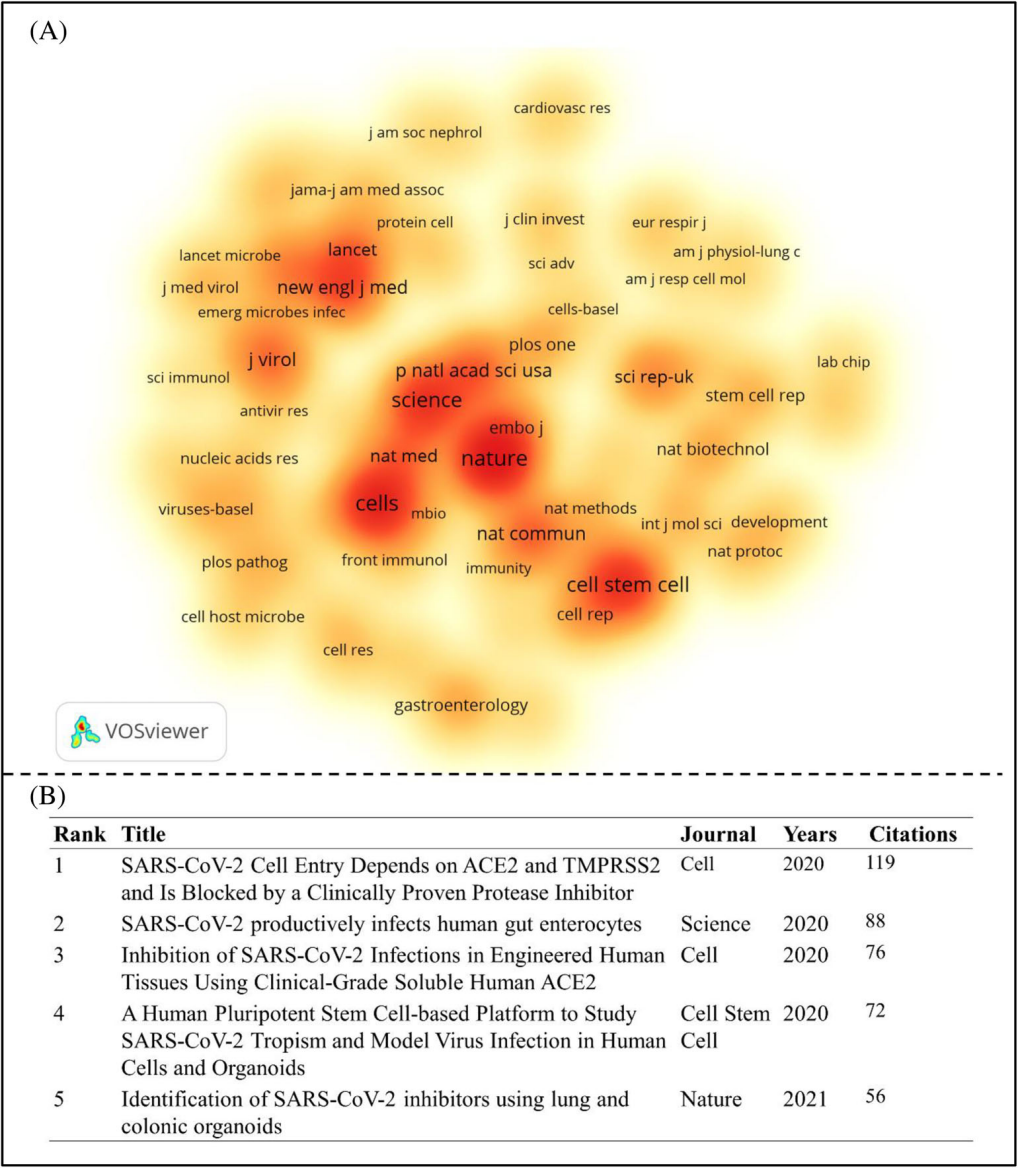
Co‐citation analysis of the publications regarding organoid application for COVID‐19 study. (A) The density visualized network of the contributing journals (more than 50 citations). The intensity of hotspots indicates the frequency of occurrences of that item. Warm colours represent hot terms and cool colours represent cool items. (B) The top 5 co‐cited references for organoid‐based COVID‐19 research.

Co‐citation analysis will be helpful for the reader to know the frequency of co‐cited references and journals and can promote the development of the disciplines.^37^, ^38^ Hence, VOSviewer was applied to visualize the corresponding journals in the field of organoid applications in COVID‐19 research. A total of 69 journals were identified with more than 50 co‐cited papers, as shown in Figure 2A. The intensity of hotspots indicates the frequency of occurrences of that item. Warm colours represent hot items and cool colours represent cool items. The top 10 contributing journals based on citations are *Nature*, *Cells*, *Science*, *Cell Stem Cell*, *New England Journal of Medicine*, *Proceedings of the National Academy of Sciences of the United States of America*, *Nature Communications*, *the Journal of Virology*, *Nature Methods* and *Lancet*. In terms of co‐cited references, the top 5 references were listed in Figure 2B. The paper ‘*SARS‐CoV‐2 Cell Entry Depends on ACE2 and TMPRSS2 and Is Blocked by a Clinically Proven Protease Inhibitor*’ (https://doi.org/10.1016/j.cell.2020.02.052) was published in *Cell* in 2020 and received the highest co‐citation (119 times) in this field. The authors provided critical insights into the early stage of SARS‐CoV‐2 infection, which depends on the host cell factor ACE2 and co‐factor TMPRSS2 (transmembrane serine protease 2), thus identified potential targets for protection against the virus.^39^ Although the study did not focus on organoids, this groundbreaking will help decipher the pathomechanism of viral infection and drug candidates screening for COVID‐19 therapeutics. The flowing four publications were closely associated with human intestinal, kidney, liver, lung and colonic organoids, respectively.^11^, ^40^, ^41^, ^42^ These data indicate that human organoids contribute to the COVID‐19 study.

### 2.5 The hotspots of in organoid‐based COVID‐19 field

**FIGURE 3:**
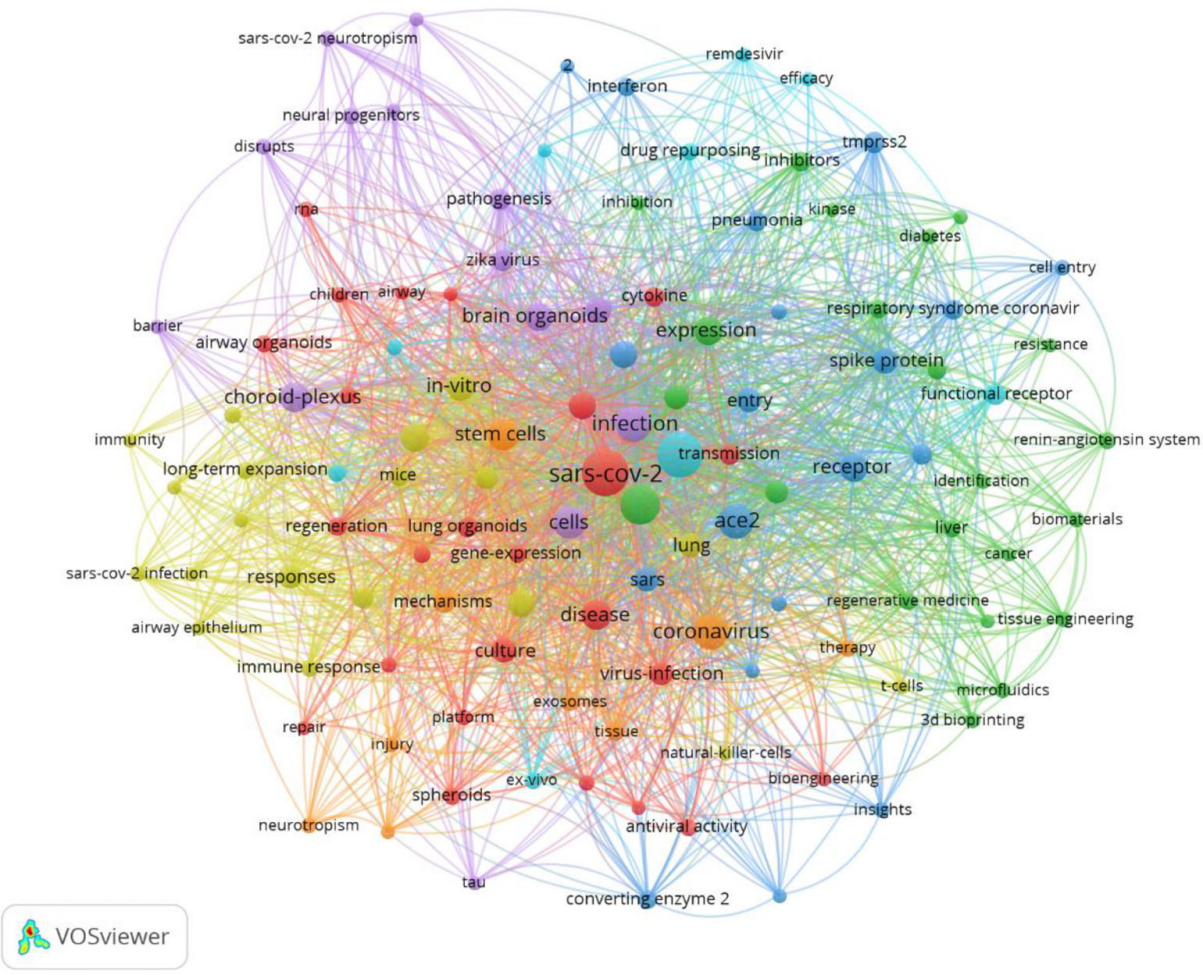
Keyword co‐occurrence (at least three times) network visualization of the research of organoids for SARS‐CoV‐2 infection. Node size indicates the occurrence frequency; node colour represents the cluster; cluster resolution = 1.00.

To better reveal the core topic in the organoid‐based COVID‐19 field, keyword co‐occurrence analysis was performed by the VOSviewer application. As shown in Figure 3, a total of 117 keywords were identified within the visualized network with a frequency of more than 3. The top 5 most frequently emerging keywords are ‘sarce‐cov‐2’ (110 co‐occurrence times), ‘covid‐19’ (99), ‘organoids’ (65), ‘ace2’ (50) and ‘infection’ (49). All 117 keywords were classified into eight clusters with distinct colours. The largest cluster, red colour, involved keywords associated with stem cells and disease models, such as ‘airway organoids’, ‘lung organoids’, ‘epithelial‐cells’, ‘transmission’, ‘disease’ and ‘virus‐infection’; Cluster 2 (green) involved in keywords associated with drug discovery and viral replication, such as ‘inhibitors’, ‘inhibition’, ‘resistance’ and ‘expression’; Cluster 3 (blue) involved in keywords associated with viral cell entry and virus–host interaction, such as ‘spike protein’, ‘entry’, ‘receptor’, ‘ace2’ and ‘tmprss2’; Cluster 4 (yellow) involved in keywords related to SARS‐CoV‐2 infection and immune response, such as ‘immunity’, ‘responses’, ‘t‐cells’ and ‘airway epithelium’; Cluster 5 (purple) involved in keywords associated with neural infection, such as ‘brain organoids’, ‘choroid‐plexus’, ‘neural progenitors’, ‘tau’ and ‘sars‐cov‐2 neurotropism’; and other clusters are associated with ‘stem cells’, ‘air–liquid interface’ and ‘intestinal organoids’.

To obtain the emergence of keywords over time, the overlay visualization of keywords regarding organoid‐based COVID‐19 research was presented in Figure S1, a derivative of Figure 3. As indicated, the node colour stands for the average publication year (purple means that keywords occurred earlier and marked with yellow stands that keywords are vigorous recently). For example, keywords, such as ‘virus‐infection’, ‘sars‐cov‐2 infection’, ‘replication’, ‘airway’, ‘airway epithelium’, ‘acute respiratory syndrome’, ‘pneumonia’ and ‘ace2’ are active in the early era. This suggests more studies focused on pulmonary infection using organoid models at the early stages of the COVID‐19 pandemic. Recently, keywords included ‘drug repurposing’, ‘personalized medicine’, ‘repair’, ‘therapy’, ‘resistance’ and ‘therapeutics’ are more popular. Therefore, more strategies are urgently demanded for SARS‐CoV‐2 therapeutics based on organoid technology.

## 3 HUMAN ORGANOIDS FOR STUDYING SARS‐CoV‐2 INFECTIVITY

Human organoids are in vitro 3D tissues formed by the self‐assembly of human stem cells. Stem cell‐derived EBs can generate derivatives of three embryonic germ layers, including ectoderm, mesoderm and endoderm and thus various organoids are generated. Given human organoids can mimic the key features of tissues and organs in the human body, such as cell diversity, structural and functional properties and physiological microenvironment, organoid platforms have been applied for studying SARS‐CoV‐2 infectivity.

### 3.1 Respiratory organoids

**FIGURE 4:**
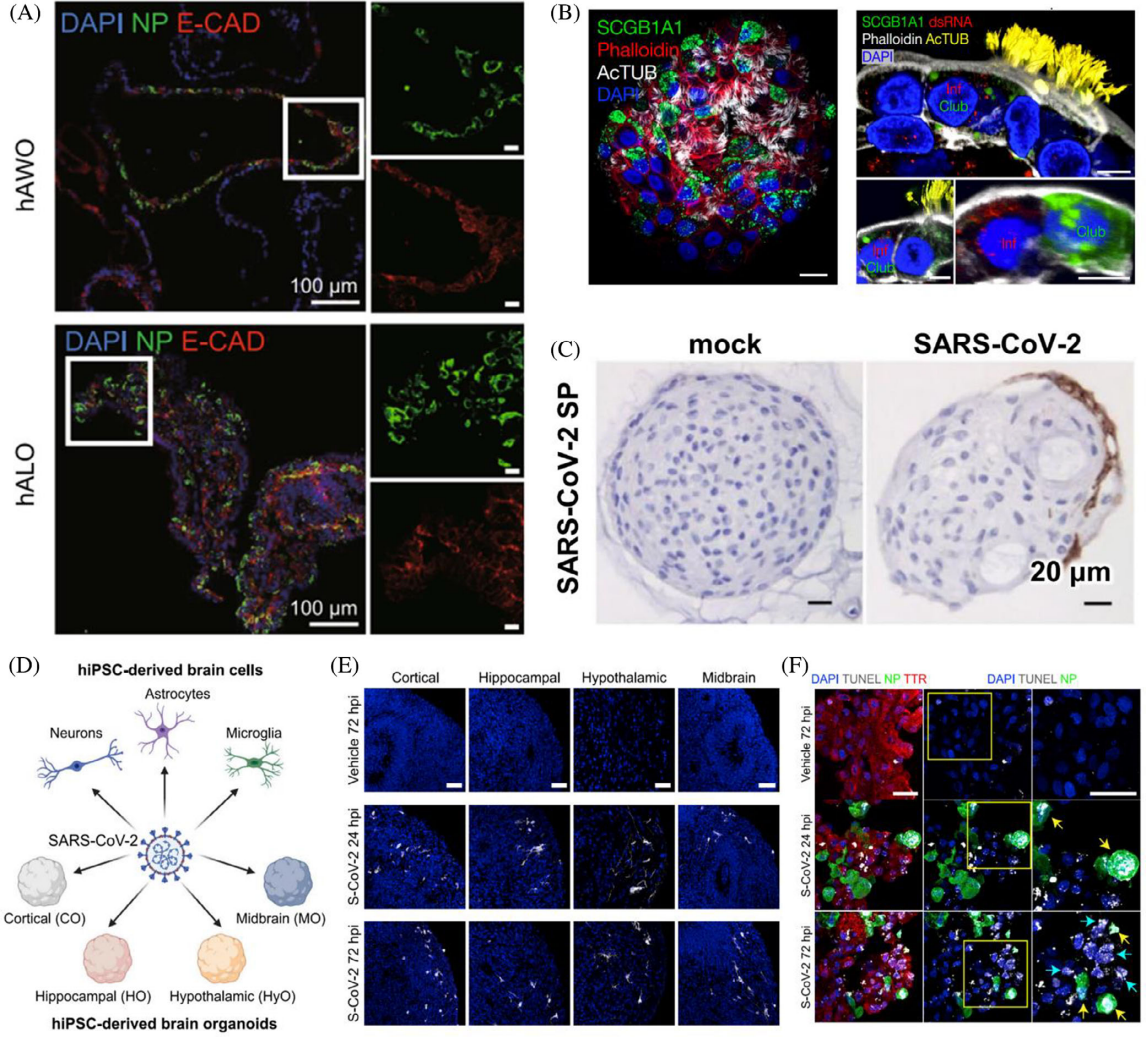
Human lung and brain organoids for modelling SARS‐CoV‐2 infection. (A) Generation of human airway organoids (hAWOs) and human alveolar organoids (hALOs) from hESCs for SARS‐CoV‐2 infection. Reproduced with permission.^46^ Copyright 2020, Oxford University Press. (B) Generation of apical‐out distal lung organoids for modelling SARS‐CoV‐2 infection. Reproduced with permission.^47^ Copyright 2020, Springer Nature. (C) Human bronchial organoids were infected by SARS‐CoV‐2. Reproduced with permission.^48^ Copyright 2022, Springer Nature. (D–F) hiPSC‐derived cortical, hippocampal, hypothalamic, midbrain organoids (D,E) and choroid plexus organoids (F) could be infected by SARS‐CoV‐2. Reproduced with permission.^49^ Copyright 2020, Elsevier.

The human airways comprise the nasal cavity, proximal and intermediate airways, respiratory bronchioles and alveoli.^43^ The airway epithelium is the primary target of SARS‐CoV‐2, which causes severe cough, excessive mucous production, shortness of breath, chest tightness and wheezing.^2^, ^44^ Studies regarding viral lung infection appeared relatively earlier in the early stages of the COVID‐19 pandemic (Figure S1). ACE2 and TMPRSS2, the key host proteins that promote cellular entry of SARS‐CoV‐2, were highly expressed on the lung epithelium.^45^ To date, nasal, bronchial, bronchioalveolar and alveolar organoids have been generated to investigate SARS‐CoV‐2 infectivity (Figure 4A–C).^2^, ^48^, ^50^, ^51^, ^52^, ^53^, ^54^, ^55^ The upper airway, particularly the nasopharyngeal epithelium, is the entry portal and primary site for SARS‐CoV‐2 replication and transmission.^51^, ^56^, ^57^ Recently, nasal epithelial cells procured via a non‐invasive procedure was used to generate 3D differentiated nasal organoids that adequately simulate the native nasal epithelium, accommodating all airway epithelial cell types, such as ciliated, basal, goblet and club cells.^51^ The differentiated nasal organoids accurately recapitulated the differential infectivety of emerging variants. Moreover, the study revealed the potentially viral pathogenesis was closely associated with ciliary damage and tight junction disruption.^51^

hPSC‐derived airway organoids (AWOs), containing functional multi‐ciliated cells, basal cells, mucus‐producing secretory cells and CC10‐secreting club cells, were also used to monitor SARS‐CoV‐2 infection, showing ciliated and club cells were infected.^46^, ^58^, ^59^ In the human lung, ciliated cells are decreased while club cells are increased from the proximal to the distal airway.^46^ Therefore, it was speculated that SARS‐CoV‐2 sequentially infected ciliated and club cells along the upper airway down to the alveoli. Moreover, SARS‐CoV‐2 infection could cause metabolic disorders, as illustrated by downregulated lipid metabolism and upregulated glycolysis.^46^, ^60^ The data of RNA‐sequence (RNA‐seq) of infected AWOs showed the upregulation of cytokine/chemokine signalling, which was consistent with exuberant inflammatory cytokine production in human COVID‐19 pulmonary infections.^60^, ^61^ In this case, AWOs thus provide a promising platform for anti‐viral therapeutic drug discovery. Moreover, human bronchial organoids (BCOs), containing transient secretory, goblet and ciliated cells, were generated for SARS‐CoV‐2 research.^48^, ^62^ The study from Sano's group proved that SARS‐CoV‐2 efficiently infected ciliated cells in BCOs, whereas not basal cells. It is probably due to ciliated cells expressing ACE2, but not basal cells, or club cells could dedifferentiate into basal cells.^48^, ^63^ The differentially expressed genes caused by viral infection were closely associated with immune response and cytokines.^64^

In addition to nasal organoids and AWOs, alveolar lung organoids (ALOs) were also used for SARS‐CoV‐2 studies. The ALO consists of type I cells (AT1) and alveolar type II cells (AT2), which functionally mimic the alveolar epithelium.^65^ hPSC‐derived ALOs have been applied to reveal the alveolar infection of SARS‐CoV‐2, showing that AT2 cells were permissive to SARS‐CoV‐2 infection.^40^, ^47^, ^66^ Given the complicated protocol and less reproducibility of PSC‐derived lung organoids, more ALOs have been established from purified primary alveolar stem cells.^67^, ^68^ For example, Salahudeen and colleagues established human distal lung organoids, including AT2 organoid and basal organoid, for exploring SARS‐CoV‐2 infection.^47^ The organoids with apical‐out polarity presenting ACE2 promoted viral infection in AT2, basal and club cells. Similarly, Chiu et al. proved that two‐dimensional (2D) ALOs sustained productive SARS‐CoV‐2, but the virus replicative fitness was lower than that in 2D AWOs.^65^ This could be due to more ACE2^+^ cells in AWOs compared to ALOs. This study suggests that higher viral transmissibility could occur in the lung airway than in the alveoli. More interestingly, it was reported that the Omicron variant only occurred in AWOs, not in ALOs.^65^ Consistently, researchers from the LKS Faculty of Medicine at the University of Hong Kong (HKUMed) proved that Omicron SARS‐CoV‐2 could infect and spread faster than ancestral strains in human bronchus but with lower severity in the lung.^69^ Another study from Chiu et al. showed that the differentiated nasal organoid monolayers accurately reproduced the highest transmissibility of the Omicron variant.^51^ Moreover, the Omicron's replicative advantage was more remarkable in the nasal organoid monolayer than that in the AWOs. This evidence suggests that nasal organoids could more adequately recapitulate the variable replication capacity and transmissibility of emerging SARS‐CoV‐2 variants than AWOs.

### 3.2 Brain organoids

Besides respiratory insufficiency, existing studies have highlighted severe neurological complications in COVID‐19 patients, ranging from headache and loss of smell to confusion and disabling strokes.^70^, ^71^ Despite viral RNA being detected in patient‐derived brain samples, it remains unclear the neurotropism of SARS‐CoV‐2 and its potential pathogenesis.^72^, ^73^, ^74^ Given the great potential to elucidate the relationship between ZIKV infection and microcephaly, brain organoids show great promise for understanding the neurotropism and neurotoxic effects of SARS‐CoV‐2.^25^, ^66^ At the early onset of the COVID‐19 pandemic, SARS‐CoV‐2 (NRW‐42) isolated from a nasopharyngeal and oropharyngeal swab specimen of infected patients was found to enter human organoids with 2‐day viral exposure but did not appear to actively replicate.^75^ The study also showed that SARA‐CoV‐2 preferred relatively mature neurons in brain organoids. For instance, mature cortical neurons in the brain organoids were susceptible to SARA‐CoV‐2 exposure.^75^, ^76^ However, this is indeed markedly inconsistent with ZIKV infection, which was found to preferentially target neural progenitor cells and trigger them to premature differentiation.^25^, ^26^, ^77^ The authors also proved that SARS‐CoV‐2 infection was related to aberrant Tau localization from axons to soma and hyperphosphorylation, which might lead to neuronal death.^75^ High‐density viral regions have more apoptotic cells compared with the low‐density viral area.^76^ Moreover, SARS‐CoV‐2 infection was accompanied by metabolic alteration in infected and neighbouring neurons, which may create a resource‐restricted environment for neural cells.

Recently, region‐specific brain organoids, including cortical, hippocampal, hypothalamic and midbrain organoids, as well as choroid‐plexus organoids (CPOs) have been established to systematically test SARS‐CoV‐2 neurotropism (Figure 4D–F).^49^, ^78^ The study from Jacob's team showed that the virus exhibited high infectivity of choroid plexus epithelial cells while limited affinity with neurons and astrocytes.^49^ The transcriptional alteration in infected CPOs showed that SARS‐CoV‐2 caused inflammatory responses and damaged the choroid plexus, indicating that the choroid plexus epithelium might be a potential gateway for viral entry to the brain and contribute to neural infection. Consistently, Pellegrini et al. proved that SARS‐CoV‐2 could infect choroid plexus epithelial cells of the brain but not neurons, leading to a potential breakdown of blood‐cerebrospinal fluid barrier integrity and development of neurological complications.^79^ This could be closely associated with neural progenitors and neurons do not specifically express SARS‐CoV‐2 entry factors or co‐factors, ACE2 and TMPRSS2.^71^, ^79^, ^80^ Conversely, in line with ZIKV infection, Zhang and colleagues proved that ACE2 and TMPRSS2 expressed in neural progenitor cells and SARS‐CoV‐2 directly infected neural progenitor cells in brain organoids.^81^ Moreover, Wang et al. demonstrated that SARS‐CoV‐2 infected hiPSC‐derived neurons, astrocytes and brain organoids, especially ApoE4 astrocytes exhibited high susceptibility to SARS‐CoV‐2.^82^ These discrepancies could be caused by the difference in differentiation protocol and differential neuronal phenotypes regulating SARS‐CoV‐2 susceptibility. These findings unambiguously demonstrate that brain organoids provide great promising platforms for probing the neurotropic features and neurotoxic effects of SARS‐CoV‐2.

### 3.3 Gastrointestinal organoids

**FIGURE 5:**
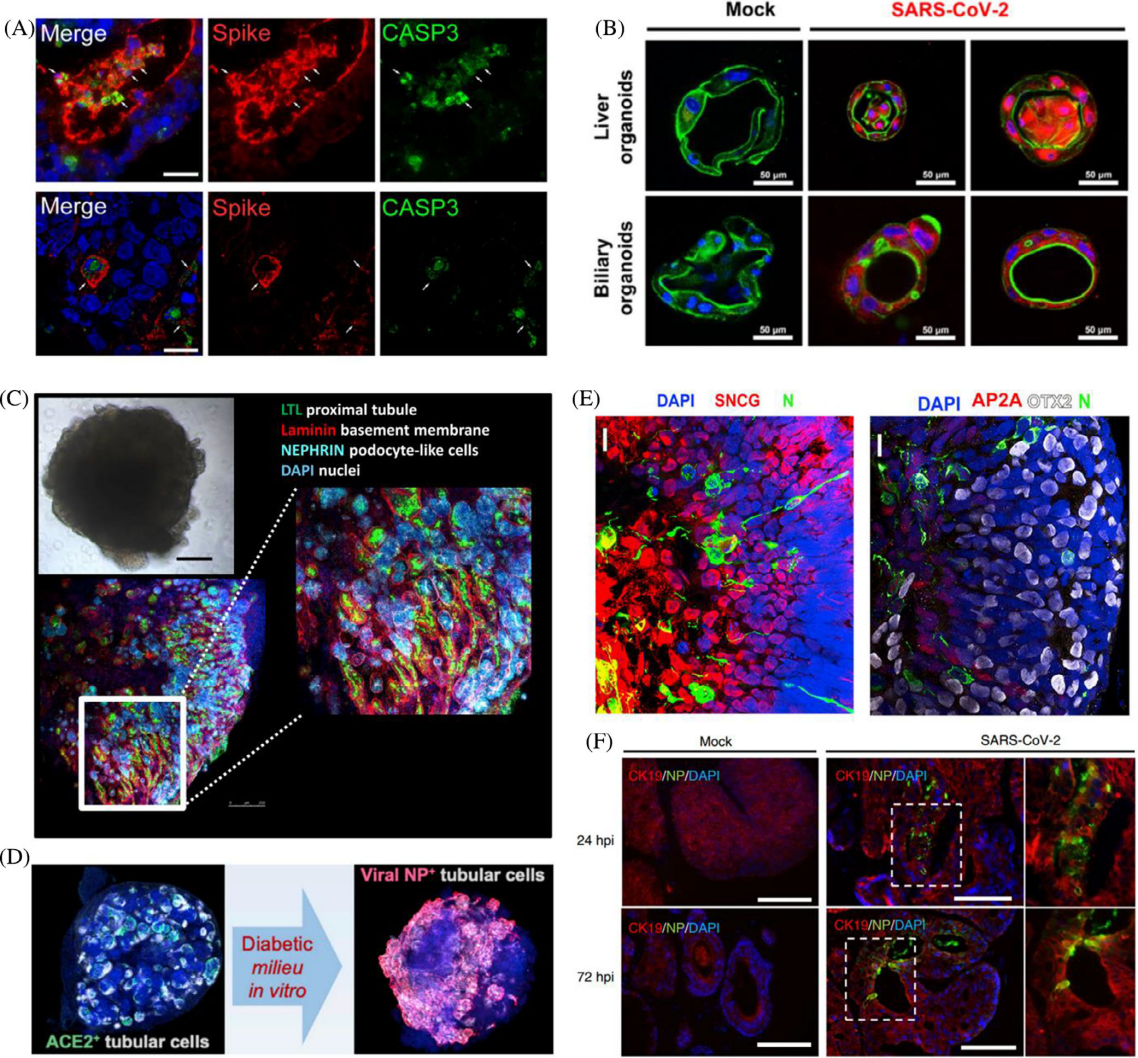
Human organoids for modelling SARS‐CoV‐2 infection. (A) Generation of human intestinal organoids for modelling SARS‐CoV‐2 infection. Reproduced with permission.^88^ Copyright 2021, Elsevier. (B) SARS‐CoV‐2 infected liver organoids and biliary organoids. Reproduced with permission.^89^ Copyright 2022, BMJ Publishing Group Ltd. (C) Generation of human kidney organoids for modelling SARS‐CoV‐2 infection. Reproduced with permission.^42^ Copyright 2020, (D) Diabetic conditions enhanced SARS‐CoV‐2 infections in human kidney organoids. Reproduced with permission.^90^ Copyright 2022, Elsevier. (E) SARS‐CoV‐2 infected and replicated in retinal organoids. Reproduced with permission.^91^ Copyright 2022, Elsevier. (F) SARS‐CoV‐2 infected salivary gland organoids. Reproduced with permission.^92^ Copyright 2022, Springer Nature.

Multiple studies have reported that COVID‐19 patients developed gastrointestinal symptoms, such as diarrhoea, vomiting, or abdominal pain,^11^, ^83^, ^84^, ^85^ suggesting that gastrointestinal tracts are considered targets of SARS‐CoV‐2. Given live virus was detected in the faeces of COVID‐19 patients, SARS‐CoV‐2 could be transmitted through the faecal‐oral route.^84^, ^86^, ^87^ Gastrointestinal organoids, such as intestinal organoids, colonic organoids and gastric organoids have been generated to unravel the susceptibility of gastrointestinal tracts to SARS‐CoV‐2 (Figure 5A).^40^, ^87^, ^88^, ^93^, ^94^, ^95^ Human and bat intestinal organoids were successfully generated by Zhou's group and first applied for modelling enteric SARS‐CoV‐2 infection.^96^ Active viral replication was found in intestinal organoids, which contain enterocytes, goblet cells, Paneth cells and enteroendocrine cells. The study showed that enterocytes were the main targets of SARS‐CoV‐2 probably due to ACE2 and TMPRSS2 being highly expressed in differentiated enterocytes. This finding is consistent with the data from Zhang's study, showing active SARS‐CoV‐2 replication in ACE2^+^ human mature enterocytes.^97^ The authors also proved that TMPRSS2 and TMPRSS4 facilitated SARS‐CoV‐2 entry into host cells by promoting viral spike fusogenic activity. Enterocytes in human colonic organoids were also sensitive to SARS‐CoV‐2 infection.^40^ Promoting enterocyte differentiation in the organoid could increase ACE2 expression.^98^ RNA‐seq analysis proved that viral infection in colonic organoids involved cytokines and chemokines signalling pathways.^40^

Apart from intestinal and colonic organoids, gastric organoids are also susceptible to SARS‐CoV‐2 infection. Giobbe et al. generated gastric organoids from fetal, pediatric and adult biopsies for modelling SARS‐CoV‐2 infection and demonstrated that late fetal and pediatric organoids allowed higher viral replication.^93^ This could be caused by the lower levels of ACE2 and TMPRSS2 in the undifferentiated organoids, including early fetal and adult organoids, compared to organoids following differentiation. Consistently, several studies showed that differentiated intestinal organoids exhibited higher susceptibility to viral infection than undifferentiated ones.^95^, ^99^ SARS‐CoV‐2 infection was also found to induce mild innate antiviral responses, particularly involved in the interferon family in late fetal and pediatric organoids as illustrated by the transcription analysis. Likewise, single‐cell RNA‐seq (scRNA‐seq) analysis of viral‐infected intestinal organoids highlighted infected cells activated strong proinflammatory events and induced interferon production.^100^ These data indicate that human gastrointestinal organoids serve as a valuable platform for SARS‐CoV‐2 gastrointestinal infection and the development of therapeutic strategies.

### 3.4 Liver organoids

As epidemiologic studies reported that COVID‐19 patients had abnormal liver function mainly involved in hepatocytes and cholangiocytes, thus human hepatocyte and cholangiocyte organoids were used to validate these findings.^101^, ^102^, ^103^ ACE2 and TMPRSS2 were expressed in hepatocytes and cholangiocytes, making cells highly vulnerable to SARS‐CoV‐2 infection.^11^, ^104^ Therefore, viral replication was observed in both hepatocyte and cholangiocyte organoids after viral infection.^11^ Meanwhile, the SARS‐CoV‐2 pseudo‐entry virus was found to infect hepatocytes. The SARS‐CoV‐2 infection activated cellular cytokine–cytokine receptor interaction, IL‐17, chemokine, TNF (tumor necrosis factor) and NF‐κB signalling pathways and downregulated metabolism in infected organoids.^11^ Similarly, Zhao et al. revealed activated TNF signalling and apoptosis pathways in the infected liver and biliary organoids, implying that SARS‐CoV‐2 infection might trigger cell death of hepatocytes and cholangiocytes (Figure 5B).^89^ A subset of human‐specific ACE2^+^/TMPRSS2^+^ cholangiocytes was identified in human liver ductal organoids, thus the organoids were extremely susceptible to SARS‐CoV‐2, leading to robust viral replication was detected in organoids.^104^ Furthermore, SARS‐CoV‐2 exposure caused the disruption of the barrier and bile acid transporting functions of cholangiocytes through the dysregulation of gene expression, which was closely associated with a tight junction formation and bile acid transportation. Therefore, SARS‐CoV‐2 infection might induce cholangiocyte injuries and consequent bile acid accumulation in COVID‐19 patients.

### 3.5 Kidney organoids

Kidney failure was also frequently observed in COVID‐19 patients.^105^ Recent studies detected viral RNA in urine samples of COVID‐19 patients.^106^ ACE2 was expressed in kidney proximal convoluted tubules, indicating that renal tubule cells could be potential targets of SARS‐CoV‐2.^107^, ^108^ Thus, 3D human kidney organoids containing proximal tubule epithelial cells were generated for SARS‐CoV‐2 infection (Figure 5C).^42^, ^109^ Compared with traditional 2D culture, ACE2 was higher expressed in organoids, indicating the organoids would be more permissive to SARS‐CoV‐2.^109^ Apart from proximal tubular cells, podocytes and stromal cells were also infected by SARS‐CoV‐2 in iPSC‐derived kidney organoids, which was confirmed by the results from the scRNA‐seq analysis.^110^ Activated profibrotic signalling was also found in infected kidney organoids and could be inhibited by a protease blocker. More recently, Garreta and colleagues established diabetic human kidney organoids that exhibited higher susceptibility to SARS‐CoV‐2 compared to non‐diabetic control (Figure 5D).^90^ This was linked to diabetic‐induced upregulation of ACE2 and metabolic programming. Therefore, kidney organoids are useful tools for investigating kidney complications for SARS‐CoV‐2 infection and looking for therapeutic drugs.

### 3.6 Other organoids

SARS‐CoV‐2 infection can cause cardiac injury and dysfunction in patients and increase the risk of mortality.^111^, ^112^ Human cardiac organoids and vascular organoids have been established to explore SARS‐CoV‐2 infectivity in the cardiovascular system.^42^, ^113^, ^114^ Mils et al. demonstrated that infected cardiac organoids could recapitulate key clinical features of diastolic malfunction in COVID‐19 patients.^115^ The study also showed that cytokine‐induced cardiac dysfunction in cardiac organoids could be attenuated by using bromodomain and extraterminal family inhibitors (BETi).

To determine the ocular involvement and potential virus–host ocular interactions, Eriksen et al. generated a whole‐eye organoid, including the retina, retinal pigment epithelium, ciliary margin, iris, lens and cornea, for SARS‐CoV‐2 infection.^116^ The study showed that higher viral replication was detected in limbus than in other ocular surface cells in eye organoids. Moreover, retinal organoids also were used to investigate retinal SARS‐CoV‐2 infection (Figure 5E).^91^, ^117^ Although relatively low expression of ACE2 in the retina, the study proved that SARS‐CoV‐2 infected retinal organoids, replicated in retinal cells, such as retinal ganglion cells and photoreceptors and induced the expression of inflammatory genes, such as interleukin 33.^91^

It was recently reported that saliva could be a potential source of SARS‐CoV‐2 transmission due to the virus being identified in the salary glands of COVID‐19 patients.^60^ Indeed, salivary glands express ACE2 and TMPRSS2 and serve as reservoirs of viruses.^60^, ^92^, ^118^ Tanaka and colleagues generated salivary gland organoids (SGOs) from hiPSCs to model SARS‐CoV‐2 infection of salary glands (Figure 5F).^92^ The human SGOs mimicked human embryonic salary gland characteristics and functions and expressed viral entry factors and co‐factors, including ACE2 and TMPRSS2. Thus, viral infection and replication were found in organoids after exposure to SARS‐CoV‐2. Although SGO is a prospective model for studying SARS‐CoV‐2 infection, it is not yet clear how SARS‐CoV‐2 replicated in salivary glands was secreted into saliva.

## 4 HUMAN ORGANOIDS FOR VACCINE DEVELOPMENT OF SARS‐COV‐2

Currently, COVID‐19 is still spreading and posing threats to widespread health, society and economy.^119^ Although, as of 30 November 2022, approximately 13 billion vaccine doses have been administered, more effective vaccines are urgently demanded to prevent further spread and keep people from contracting the virus.^1^, ^120^, ^121^ The development of a novel vaccine is still a lengthy process, including the initial vaccine design, preclinical testing in animal models and clinical trials (phases I–IV), which could take 15 years or more, great efforts have been paid in vaccine development responding to the COVID‐19 pandemic.^121^, ^122^ According to the WHO, as of 6 December 2022, 175 candidate vaccines are under clinical testing for treating COVID‐19 and 199 candidate vaccines are in pre‐clinical studying.^123^ Of the 175 candidates, 49 and 11 are undergoing further validations of safety and efficacy in phase III and phase IV clinical trials with a large number of volunteers, respectively. The technology platforms can be divided into traditional whole virus vaccines (inactivated or live attenuated vaccines), recombinant protein‐based vaccines (protein subunit vaccines, virus‐like particles), viral vector vaccines and nucleic acid vaccines (DNA‐ and RNA‐based vaccines). Among them, protein subunits and RNA are mainly candidate vaccines.^123^

As viral infections are the prototypic species‐specific diseases and pronounced species‐specific differences in adaptive immunity, many candidate vaccines that worked in animals while failing in human trails.^124^, ^125^, ^126^, ^127^ Therefore, novel animal‐free test methods may accelerate the testing programs for drug and vaccine development.^124^ Although in vitro human organoids are devoid of the host immune system, organoid‐based assays could provide unique opportunities for studying the host–virus interaction and testing the efficacy and neutralizing antibodies of candidate vaccines.^40^ Several studies have proved that antibodies were effective in neutralizing SARS‐CoV‐2. For instance, Pei and colleagues evaluated the inhibitory effect of a neutralizing antibody CB6 on SARS‐CoV‐2 infection in human lung organoids.^46^ The study showed that CB6 notably suppressed viral replication in infected lung organoids. This was consistent with the previous study that reported CB6 repressed SARS‐CoV‐2 infection in rhesus monkeys.^128^ Moreover, a new tetravalent neutralizing antibody (15033‐7) and a synthetic dipeptidyl peptidase‐4 (DPP4) peptide were observed to reduce SARS‐CoV‐2 entry in human lung organoids and modulate innate immunity and inflammatory response.^129^ These data will further aid vaccine and drug development for COVID‐19. Advanced engineering approaches, such as bioreactor or chip‐based engineering devices, showing great promise to generate immune cell organoids, but they cannot recapitulate the complex of human adaptive immunity.^130^, ^131^, ^132^ Importantly, the presence of immune elements, including B cells, T cells and microphages in organoid models might facilitate vaccine development.^40^

**FIGURE 6:**
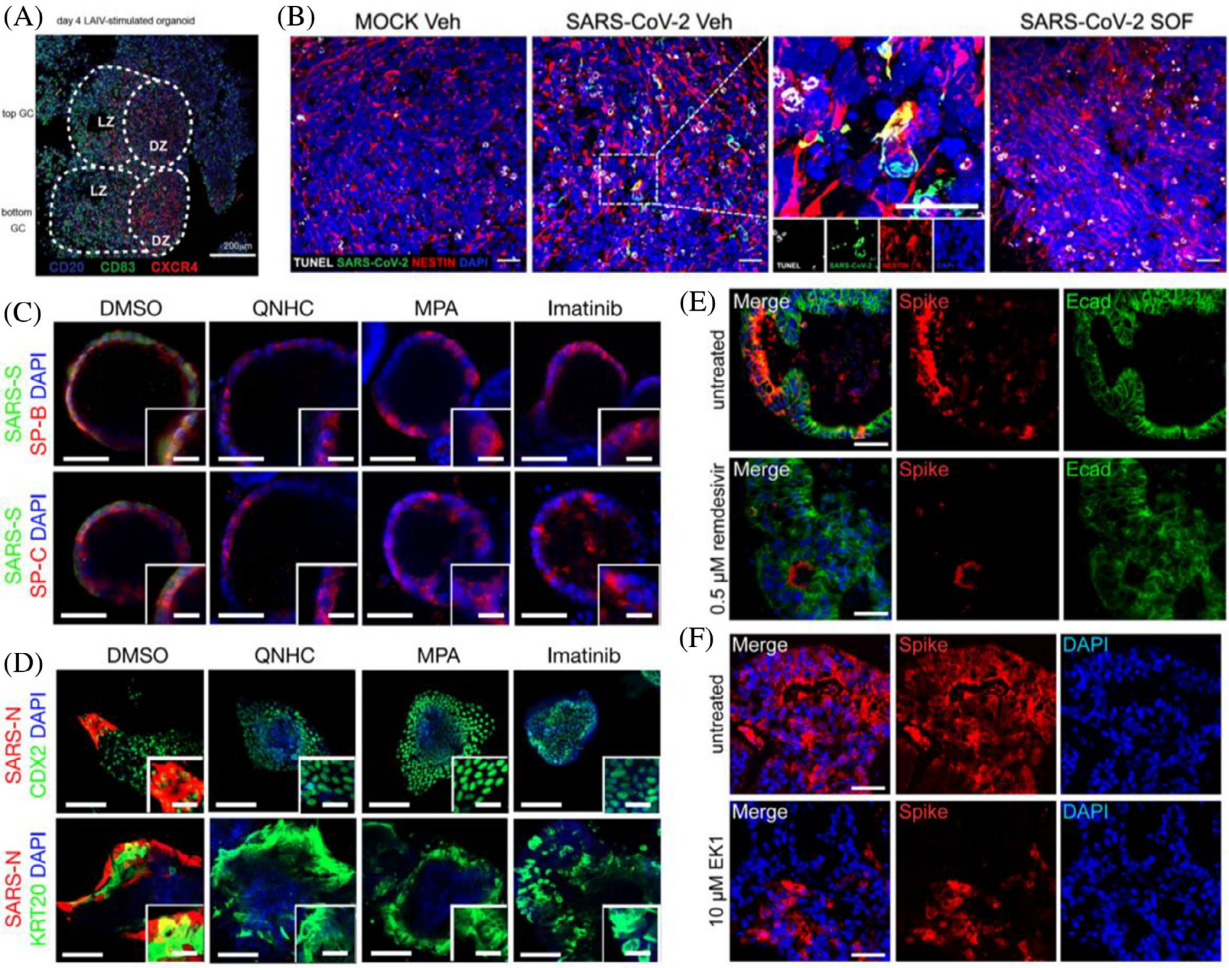
Human organoids for vaccine development and drug discovery of SARS‐CoV‐2. (A) Generation of human tonsil organoids for SARS‐CoV‐2 infection and vaccine development. Reproduced with permission.^126^ Copyright 2021, Springer Nature. (B) SOF (sofosbuvir) could inhibit SARS‐CoV‐2 replication in brain cells and rescue neurological impairments in human brain organoids. Reproduced with permission.^133^ Copyright 2022, Public Library of Science. (C,D) QNHC (quinacrine dihydrochloride), imatinib, and MPA (mycophenolic acid) blocked the entry of SARS‐CoV‐2 in both hPSC‐derived lung organoids (C) and colonic organoids (D). Reproduced with permission.^40^ Copyright 2021, Springer Nature. (E,F) EK1 and remdesivir inhibited infection of human intestinal organoids with SARS‐CoV‐2. Reproduced with permission.^88^ Copyright 2021, Elsevier.

Recently, Wagar et al. established human tonsil organoids (TOs) from reaggregating the dissociated primary human tonsils to evaluate human adaptive immune responses to the SARS‐CoV‐2 vaccine.^126^ The specialized germinal centre (GC)‐like structure with distinct T cells and B cells was identified in human TOs, which is essential to produce antigen‐specific antibodies, affinity maturation, B cell differentiation and class‐switch recombination (Figure 6A). Increased B cell differentiation and influenza‐specific antibody production were observed in organoids after exposure to live attenuated influenza vaccine. To clear the responses of individual cells to influenza, the authors analysed the changes in plasmablast differentiation and antigen‐specific antibody production after antigen‐presenting cell (APC), T cell and B cell subsets depletion. The results showed that naive B cells, APCs, CD4^+^, or CD45^−^ stromal cells were minimally needed to constantly maintain plasmablast differentiation and naive antibody responses.^126^ Subsequently, TOs from different donors were used to investigate the response to the adenovirus‐based SARS‐CoV‐2 vaccine. Increased plasmablast differentiation, CD8^+^ T cell activation, and IgG and IgA antibody production were observed in TOs after 14‐day stimulation. Therefore, human TO application in COVID‐19 could help decipher immune responses to viruses and accelerate vaccine design.^134^ Although this model was not lacking in immune components, it is worth noting that human organoids used for viral vaccine development are in isolation. The organoid model could not well mimic the immune cell migration from pathogen entry sites to the lymph nodes, tonsils, or spleen, where GCs form in humans.^126^ Future studies are needed to incorporate more methods to capture features of human adaptive responses. Moreover, safety considerations are the most important things for vaccine development.^120^ It remains to be seen whether new SARS‐CoV‐2 vaccines could trigger aberrant immune responses and induce potential multi‐organ injuries.

## 5 HUMAN ORGANOIDS FOR DRUG DISCOVERY OF SARS‐COV‐2

Given traditional cell lines cannot recapitulate the physiologically relevant kinetics of in vivo SARS‐CoV‐2 infection, human organoids provide promising models for drug discovery of COVID‐19. The respiratory system is the initial viral target, thus human lung organoids were used to test the efficacy of drugs and compounds, focusing on viral entry and replication.^135^, ^136^ For instance, Xu et al. demonstrated that inhibition of receptor‐interacting serine/threonine‐protein kinase 1 (RIPK1 kinase) caused by RIPK1 inhibitor Nec‐1s reduced viral load and inflammatory responses in infected lung organoids.^137^ The results suggest that the function of RIPK1 in SARS‐CoV‐2 propagation and suppression of RIPK1 might provide a strategy for COVID‐19 therapeutics and prevention. Since ACE2 serves as the entry receptor, a humanized decoy antibody (ACE2‐Fc fusion protein) has been designed to target the interaction between ACE2 and viral spike protein.^138^ The study proved that ACE2‐Fc effectively blocked the entry of SARS‐CoV‐2 spike‐expressing pseudotyped virus into lung organoids, thus suppressing viral replication, suggesting that ACE2‐Fc provides a latent prospect for COVID‐19 treatment. Samuel and colleagues demonstrated that androgen (AR) signalling regulated the entry factors and co‐factors of SARS‐CoV‐2 directly, ACE2 in addition to TMPRSS2.^139^ Thus, drugs inhibiting AR signalling were found to reduce viral infection in hESC‐derived lung organoids. The study underscores the critical roles of AR signalling in viral infection and lays the foundation for antiandrogenic drug application in COVID‐19 therapeutics.

Primary human lung epithelial infection models, consisting of differentiated air–liquid interface (ALI) cultures of proximal airway epithelium and alveosphere cultures of distal lung AT2 cells, were applied to validate the efficacy of remdesivir, hydroxychloroquine, and interferon beta 1 (IFN‐β1) in anti‐SARS‐CoV‐2.^140^ The results confirmed that three molecules significantly suppressed the infection and replication of SARS‐CoV‐2. Remdesivir, an inhibitor of the viral RNA‐dependent, is the most promising FDA‐approved drug in clinical use for hospitalized COVID‐19 patients.^128^, ^141^ Treatment of remdesivir inhibited viral replication and alleviated alveolar‐capillary barrier disruption on bioengineered human alveolus chip.^142^ Similarly, Pei et al. demonstrated remdesivir inhibited viral production in AWO and ALO models.^46^ Since the role of interferon lambda (IFN‐λ) in the innate antiviral immunity in the respiratory tract, Lamers and colleagues proved that SARS‐CoV‐2 replication and dissemination were abrogated by low‐dose IFN‐λ1 treatment in the organoid‐derived bronchioalveolar model.^54^ The study from Katsura's group also proved that pre‐treatment with a low dose of IFN alpha (IFN‐α) and IFN gamma (IFN‐γ) blocked SARS‐CoV‐2 replication in alveospheres.^67^ Moreover, pre‐treatment with both IFN‐λ and IFN‐β1 was observed to significantly impaired SARS‐CoV‐2 infection in colonic organoids.^143^ These data indicate interferons will be a viable therapeutic option for COVID‐19.^144^, ^145^

**FIGURE 7:**
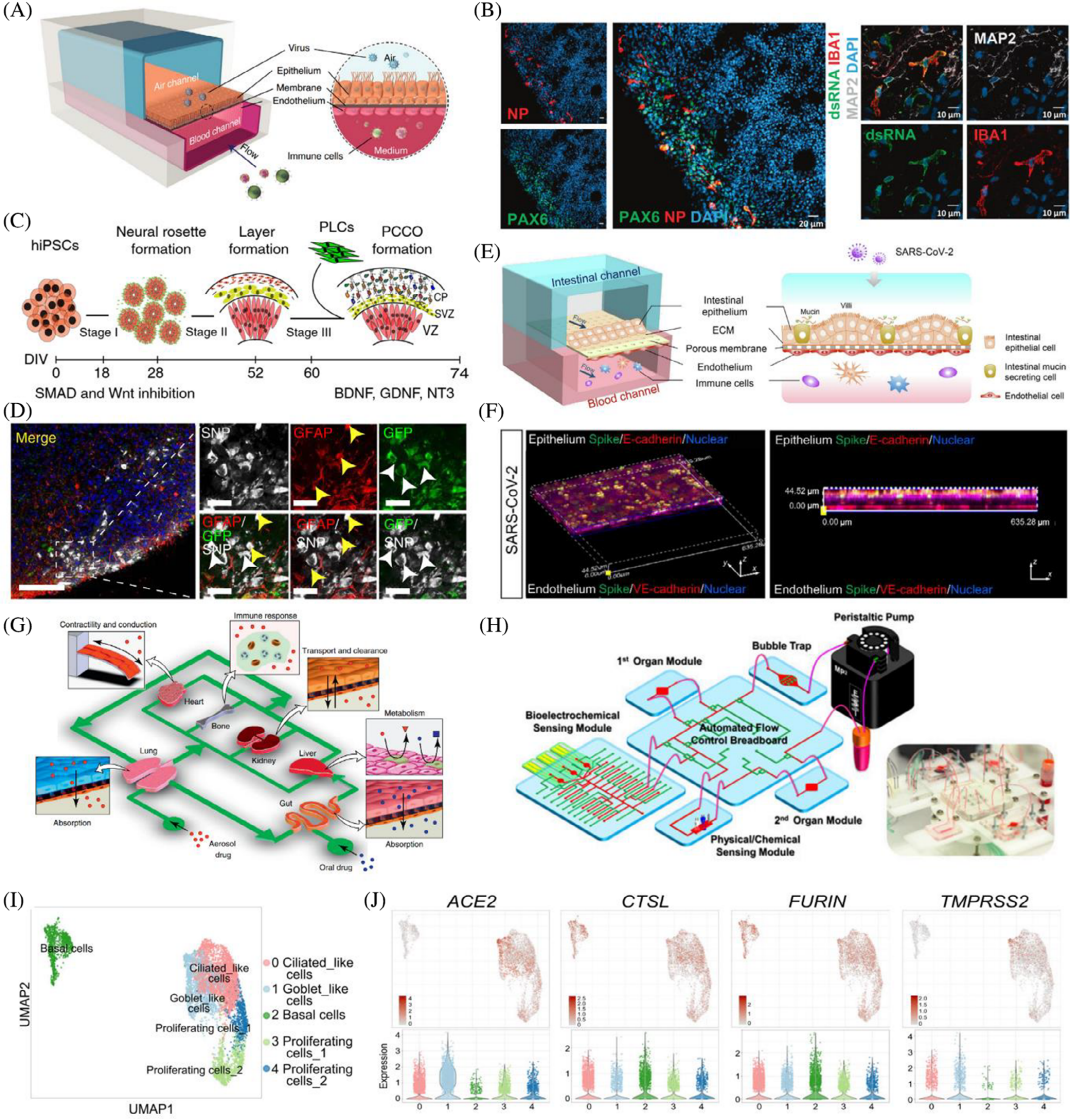
Building advanced human organoids by integrating novel strategies and technologies. (A) A human‐airway‐on‐a‐chip for studying viral infection. Reproduced with permission.^146^ Copyright 2021, Springer Nature. (B) SARS‐CoV‐2 infection in brain organoids with innately developing microglia. Reproduced with permission.^147^ Copyright 2022, Springer Nature. (C,D) Pericyte‐like cell‐containing cortical organoids were generated by the integration of pericyte‐like cells into cortical brain organoids and were prone to be infected by SARS‐CoV‐2. Reproduced with permission.^148^ Copyright 2021, Springer Nature. (E,F) SARS‐CoV‐2 induced intestinal responses with a biomimetic human gut‐on‐chip. Reproduced with permission.^149^ Copyright 2021, Science China Press. (G) The human multi‐organ‐on‐a‐chip concept. Reproduced with permission.^150^ Copyright 2011, Elsevier. (H). Generation multisensor‐integrated organs‐on‐chips platform. Reproduced with permission.^151^ Copyright 2017, National Academy of Sciences. (I,J). Single‐cell RNA‐seq analysis of hPSC‐derived airway organoids. Reproduced with permission.^60^ Copyright 2021, Elsevier.

Human airway microfluidic devices, containing two parallel microchannels, lung bronchial‐airway basal stem cells located in an ‘airway channel’ exposed to air and lung endothelium cultured on the opposite side (‘vascular channel’) exposed to continuous fluid perfusion, have been established to test the efficacy of antiviral drug candidates (Figure 7A).^146^ The airway chip faithfully recapitulated key features of influenza‐induced human lung responses and the anti‐influenza efficacy of Oseltamivir. Moreover, the ‘airway channel’ of the airway chip was exposed to SARS‐CoV‐2 pseudoparticles to simulate viral airborne infection, confirming that SARS‐CoV‐2 pseudoparticles could infect human airway epithelial cells efficiently. Subsequently, the ‘vascular channel’ was continuously perfused by candidate drugs to simulate circulatory distribution after oral administration, and the results showed that amodiaquine and its metabolite inhibited SARS‐CoV‐2 infection.^146^ Although amodiaquine is used to prevent and treat malaria, this medicine showed great potential in antiviral therapeutics and prophylactics.

hPSC‐derived lung and colon organoids were also applied for high‐throughput screening FDA‐approved drugs, such as imatinib, mycophenolic acid, and quinacrine dihydrochloride (Figure 6C,D).^40^ The study showed that all three drugs could inhibit SARS‐CoV‐2 infection in lung and colon organoids in a dose‐dependent manner. RNA‐seq data highlighted that imatinib‐treated infected organoids were closely associated with lipid metabolism. Tiwari et al. found that EK1 peptide (spike protein inhibitor) and camostat/nafamostat (TMPRSS2 inhibitors) could block SARS‐CoV‐2 entry into lung organoids.^66^ This result was consistent with previous studies, showing the antiviral efficacy of EK1 and camostat mesylate.^39^, ^152^ Pei et al. also proved camostat exerted a slight suppressive effect on viral infection in AWOs, but not in ALOs.^46^ A recent study reported that human lung organoids were also applied to explore the antiviral activity of CH25H.^153^ Mechanistically, CH25H converted cholesterol to 25‐hydroxylase (25HC), triggering cholesterol depletion on the plasma membrane and affecting virus–cell membrane fusion, resulting in inhibiting viral entry. As 25HC is a natural product without toxicity at effective concentrations, it provides a potential antiviral agent to fight against SARS‐CoV‐2 infection.

As SARS‐CoV‐2‐infected intestinal organoids exhibited the disruption of intestinal integrity and organoid deterioration, mimicking SARS‐CoV‐2‐induced gastrointestinal damage, intestinal organoids were used to test drug efficacy in COVID‐19 patients.^88^ A study has shown that remdesivir could effectively inhibit SARS‐CoV‐2 infection dose‐dependently at low micromolar concentrations. However, famotidine did not impact viral infection and spread in intestinal organoids, which was inconsistent with a previous retrospective cohort study that proved famotidine use would be related to alleviative clinical outcomes in hospitalized COVID‐19 patients.^154^ Similar to lung organoids, EK1 peptide could inhibit viral infection of intestinal organoids (Figure 6E,F).^88^ Interferon‐inducible transmembrane (IFITM) proteins are highly expressed in intestinal organoids and function as entry co‐factors for efficient SARS‐CoV‐2 infection.^155^ Thus, IFITM‐derived peptides or antibodies targeting the N‐terminus of IFITMs were found to strongly reduce viral entry and replication in gut organoids.

A recent study showed that ACE2 expression was regulated by the farnesoid X receptor (FXR) signalling in the gastrointestinal and respiratory systems.^156^ Therefore, suppression of the FXR signalling by the off‐patent drug ursodeoxycholic acid or the over‐the‐counter compound z‐guggulsterone downregulated the expression of ACE2, subsequently reducing susceptibility to SARS‐CoV‐2 infection in cholangiocyte, airway, and intestinal organoids. The study identified a new regulation of ACE2 through the FXR signalling and provided a new therapeutic target for COVID‐19. More recently, clinical‐grade human recombinant soluble ACE2 (hrsACE2) has been found to inhibit SARS‐CoV‐2 infections of human kidney and capillary organoids dose‐dependently.^42^ Similarly, Wysocki et al. also proved that a novel soluble ACE2 variant, consisting of 618 amino acids fused with an albumin binding domain (ABD), could neutralize SARS‐CoV‐2 in human kidney organoids.^157^ More intriguingly, Monteil and colleagues studied the additive effects of combination treatment with hrsACE2 and remdesivir on infected kidney organoids.^158^ The data showed that combining both drugs at low doses caused a notably reduced viral production. This finding lays the foundations for combinatorial regimes in future COVID‐19 clinical trials. MEDS433, a new inhibitor of the human dihydroorotate dehydrogenase, also exhibited a high potency of anti‐SARS‐CoV‐2 activity in hESC‐derived kidney organoids.^159^

A study conducted by Mesci's group showed that SARS‐CoV‐2 infected brain organoids and caused neuron death, which was accompanied by excitatory synapses in neurons.^133^ SARS‐CoV‐2 regulated specific gene expression in neurons implicated in antigen presentation, viral entry via the endocytic pathway, neuronal projection development, oxidative stress, and the complement pathway. Treatment with sofosbuvir, a FDA‐approved antiviral drug, efficiently repressed SARS‐CoV‐2 replication and rescued viral‐induced neuronal damage in infected brain organoids (Figure 6B).^133^ In addition to lung and intestinal organoids, remdesivir was also found to effectively suppress SARS‐CoV‐2 infection and rescue disease phenotypes in neurons and astrocytes.^82^ Therefore, brain organoids also provide invaluable in vitro tools for COVID‐19 drug discovery and validation. Moreover, the therapeutic efficacy of remdesivir against SARS‐CoV‐2 was also validated in the tonsil organoid model.^160^

## 6 CHALLENGES AND FUTURE CONSIDERATIONS

Although human organoids provide valuable platforms for deciphering the pathology of SRAS‐CoV‐2 infection and looking for effective vaccines and potential treatments, these models still have several problems that need to be addressed. One major remaining challenge is the absence of cellular complexity and cell–cell communications, thus organoid microenvironment cannot mimic in vivo organ situations because of lacking vascular, neural, and immune cells. COVID‐19 caused by SARS‐COV‐2 infection could induce multiple organ failure, whereas conventional organoid models are devoid of physiological relevance and organ‐organ interaction.^161^, ^162^ Therefore, advanced methods and technologies are urgently needed to improve organoid advancement.

### 6.1 Immune vascular organoids

The host immune system plays a pivotal role in anti‐SARS‐CoV‐2.^163^ Given most organoids lack immune cells, human organoids co‐culturing with immune cells have been applied to investigate the pathophysiology of COVID‐19 involved with immune responses. Recently, Zhang's group established a biomimetic human alveolus chip to recapitulate the alveolar‐capillary barrier by coculture of the alveolar epithelial cells, microvascular endothelial cells, and circulating immune cells with perfusing media flow.^142^ Notably, SARS‐CoV‐2 caused immune cell recruitment and endothelium detachment and promoted inflammation, indicating the critical roles of immune cells in SARS‐CoV‐2‐induced lung alveolar‐capillary barrier injury and inflammatory response. Another study generated an immune‐cardiac coculture system containing cardiomyocytes and macrophages to study macrophage‐mediated host cell responses to SARS‐CoV‐2 infection.^164^ The results proved that macrophages induced reactive oxygen species production and apoptosis in cardiomyocytes after viral exposure. In this case, ranolazine and tofacitinib were then found to alleviate the macrophage‐induced cardiotoxicity. As astrocytes are permissive to SARS‐CoV‐2, brain organoids co‐culturing with astrocytes might boost the viral infection rate.^82^ Microglia, the resident immune cells of the central nervous system, play vital roles in brain development, homeostasis, and diseases, incorporating microglia into neural organoids provides unprecedented opportunities to study infective neurological disorders.^165^, ^166^, ^167^, ^168^, ^169^ For instance, upon ZIKV infection, microglia became activated and targeted neurons and excessively pruned synapses in microglia‐containing brain organoids.^168^ Similarly, Samudyata et al. established a protocol for generating a brain organoid model with innately developing microglia for studying SARS‐CoV‐2 infection (Figure 7B).^147^ The authors proved that viral infection induced neuronal cell death and increased microglial engulfment of postsynaptic termini in infected microglia‐containing brain organoids. ScRNA‐seq analysis showed that upregulation of interferon‐responsive genes could promote microglia‐mediated synapse elimination secondary to SARS‐CoV‐2 exposure, subsequently causing disruption in circuit integrity, leading to potential cognitive impairments. The data indicate that microglia contribute to SARS‐CoV‐2‐induced neural damage.^147^, ^170^, ^171^, ^172^ Thus, microglia‐containing brain organoid models may provide an opportunity for studying SARS‐CoV‐2‐induced neuronal damage and preventing COVID‐19 neurological sequelae.

Notably, vascular networks play critical roles in organogenesis and organ identity.^173^, ^174^ Currently, evidence is accumulating regarding how to generate vascularized human organoids.^175^, ^176^, ^177^ Therefore, human organoid vascularization would promote their applications in studying SARS‐CoV‐2 infection. For instance, as brain pericytes are proposed as SARS‐CoV‐2 infection points, Wand et al. established pericyte‐like cell (PLC)‐containing cortical organoid (PCCO) ‘assembloid’ through the integration of PLCs into cortical organoids to model SARS‐CoV‐2‐induced neuropathology (Figure 7C,D).^148^ PLCs were found to promote astrocytic maturation and capture the features of PLC–basement membrane–astrocyte structure, mimicking human brain pericyte functions in vivo. Compared with conventional cortical organoids, PCCOs exhibited higher susceptibility to SARS‐CoV‐2 due to PLCs within PCCOs serving as viral ‘replication hubs’, promoting virus cell‐to‐cell spread.^148^ The study indicates that incorporating neurovascular unit components with brain organoids could provide a promising platform for modelling neural infection of SARS‐CoV‐2. As ACE2 is expressed throughout the vasculature of the body, virus–host interactions on the vascular barrier could contribute to virus access to multiple organ systems.^178^, ^179^ To date, 3D microfluidic chips modelling the human blood–brain barrier,^179^ respiratory vascular barrier,^180^ and intestinal epithelium–vascular endothelium barrier (Figure 7E, F)^149^ have been established to investigate the effect of SARS‐CoV‐2 on the endothelial barrier. Consistently, SARS‐CoV‐2 was found to disrupt vascular endothelial adherent junctions.

### 6.2 Multi‐organoids on a chip

In recent years, the microphysiological system, named the ‘human‐on‐a‐chip’ platform, provides specific compartments for multiple organoid interconnections through microchannels (Figure 7G).^150^, ^181^ The fluidic flow in the chip is analogous to the vasculature through the organ and tissue residence time.^182^, ^183^ Compared with ‘single‐organoid‐on‐a‐chip’ (single‐OoC) models, ‘muti‐organoid‐on‐a‐chip’ (multi‐OoC) models present advantages in providing a systemic approach to decipher cross‐organ communications.^184^ Multi‐OoC platforms could be classified into two groups, namely coupling of single‐OoC units or integration of multiple organoids into one dish. However, the latter offers higher throughput and is more valuable in identifying potential biomarkers and therapeutic targets when compared to the former.^184^ Multi‐OoCs hold great promise for systemic assessment of SARS‐CoV‐2 infection, determining organ susceptibility, and looking for anti‐viral therapeutic drugs. Indeed, SARS‐CoV‐2 infection presents multi‐organ injuries and acts at a high level of organ‐organ interaction.^161^, ^162^ Therefore, a multi‐organoid system could help to study the inter‐organ communications within organs after SARS‐CoV‐2 infection and the secondary effects of the infected organ on another organ. Since multi‐OoCs have been widely applied for toxicity assessment, thus multi‐OoC platforms could be used for the safety evaluation of COVID‐19 vaccines and drugs.^24^, ^185^ Moreover, muti‐OoCs provide promising models to evaluate the complex process of drug absorption (intestine), organ distribution (along with blood circulation), drug metabolism (liver), and excretion (kidney), as well as potentially reduce the cost of preclinical animal models before conducting clinical trials.

Although the muti‐OoC system has shown enormous potential as an advanced organoid model, muti‐OoC technology is still in its infancy and maintains scientific and technical challenges. First, it is important to optimize the culture medium for co‐culturing different organoids due to tissue‐specific small molecule and growth factor requirements through the differentiation process in guided stem cell differentiation. The culture medium should be provided to meet the needs of each organoid. Noteworthy, the flow rate of the medium needs to be optimized to ensure that sufficient nutrients and oxygen meet the requirements of the next organoids. Moreover, it is necessary to consider that the metabolites generated by the organoid could be toxic to other organoids through the distribution of flow.^186^ Therefore, the OoC platform needs a sophisticated programme for nutrient‐waste management. Second, highly integrated monitoring platforms could be used to real‐time monitor cellular activity and organoid behaviours, such as cell vitality, metabolites, inflammatory cytokines, and electrophysiology.^187^, ^188^ Multisensor‐integrated OoC platform will enhance their performance in drug screening by providing biomarker profiling (Figure 7H).^151^ To overcome the high variability of organoids, multi‐organoids derived from the same hiPSC lines could resolve this problem and provide opportunities for an accurate model for personalized prediction and treatment.^181^ To date, further improvements are still demanded until multi‐OoCs become a standard COVID‐19 evaluation tool.

### 6.3 Multi‐omics analysis

The increased resolution of scRNA‐seq technologies has led to great breakthroughs and improved our understanding of cellular antiviral responses, such as hepatitis B virus (HBV),^189^, ^190^ Ebola virus,^191^ influenza virus,^192^, ^193^ rabies virus,^194^ human immunodeficiency virus (HIV),^195^, ^196^ ZIKV,^197^, ^198^, ^199^ and SARS‐CoV‐2.^189^, ^200^, ^201^, ^202^, ^203^, ^204^, ^205^, ^206^, ^207^ scRNA‐seq enables transcriptome‐wide expression profile at a single‐cell resolution and provides a powerful method to investigate virus–host and host–host interactions and intercellular communication networks upon viral infection.^208^, ^209^, ^210^ Previous evidence has shown that early scRNA‐seq technology has been applied to investigate the expression profiling of the SARS‐CoV‐2 entry factors and co‐factors, such as *ACE2*, *TMPRSS2* and *FURIN*.^45^, ^211^, ^212^, ^213^ As human organoids contain multiple differentiated cell types and allow to explore cell–cell interactions, organoid platforms combined with scRNA‐seq can be used to identify the gene expression of entry factors and co‐factors in diverse cell phenotypes.^214^, ^215^ For instance, Duan and colleagues proved that scRNA‐seq analysis validated that *ACE2*, *TMPRSS2*, and *FURIN* were expressed in the ciliated‐like cells, allowing SARS‐CoV‐2 entry in AWOs (Figure 7IJ).^60^ Similarly, the results of scRNA‐seq also showed that *ACE2*, *TMPRSS2*, and *FURIN* were enriched in the AT2‐like cell population in lung organoids.^40^ It is worth noting that the data from scRNA‐seq require to be validated by immunostaining and flow cytometry analyses. Although scRNA‐seq has gained tremendous attention, challenges in high sample preparation cost, severe batch effects, time‐consuming process, numerous sample requirements, and relatively low‐throughout manners limited the widespread adoption and scope of scRNA‐seq.^210^, ^216^ The existing strategies for sample multiplexing for scRNA‐seq with DNA‐based barcoding will help address these limitations.^217^, ^218^ Multiplexing of samples in scRNA‐seq in combination with human organoids could be successfully applied to study viral pathogenesis and develop viral vaccines and antiviral therapeutics.

In addition to transcriptomic analysis, epigenomic, proteomic, and metabolic analyses are needed to be integrated into exploring the wide dysregulation of tissues or organs upon SARS‐CoV‐2 infection.^219^, ^220^, ^221^ The paired analyses empowered the study's ability to comprehensively reveal potential mechanisms and regulatory networks that respond to SARS‐CoV‐2 infection. More recently, novel single‐cell multi‐omics have been used to investigate to better understand SARS‐CoV‐2‐induced immune responses.^222^, ^223^, ^224^, ^225^, ^226^ For instance, scRNA‐seq, CITE‐seq (cellular indexing of transcriptomes and epitopes sequencing), TCR‐seq (T cell receptor sequencing) and BCR‐seq (B cell receptor sequencing) have been utilized to illustrate the dynamic responses of the innate and adaptive immune systems to SARS‐CoV‐2.^227^ The high‐throughput immune profiling revealed progressive COVID‐19 was associated with dyssynchrony of the innate and adaptive immune responses. Similarly, Wilk's lab performed scRNA‐seq, scATAC‐seq (single‐cell assay for transposase‐accessible chromatin sequencing), and CyTOF (cytometry by time of flight) on the COVID‐19 patient‐derived peripheral immune cells.^221^ The epigenomic, proteomic and transcriptomic profiling revealed the aberrant peripheral immune responses to SARS‐CoV‐2, including neutrophil and natural killer cell hyperactivation. Regarding these, we foresee that multi‐omics technology integrated with organoid models will provide large‐scale information and improve the understanding of virus–host interactions.

## 7 CONCLUSION REMARKS

The present review aims to reveal the status and research trend of organoid applications systematically and comprehensively for COVID‐19 research, including studying the infection mechanism, vaccine development and drug discovery of SARS‐CoV‐2. Although fruitful data have been obtained, it cannot be denied that the organoid‐based COVID‐19 study is still in its infancy. Human organoids are closely resembling embryonic or fetal organs rather than mature adult organs. It remains to be seen whether organoid maturation could cause diverse cellular responses and susceptibility to viral infections between immature and mature organs. Moreover, differences in different organoid protocols might induce diverse susceptibility to SARS‐CoV‐2 infection. Building advanced human organoids by integrating novel strategies and technologies could address the challenges and promote the development of COVID‐19 research. We hope this study could fully present the status and research trend of organoid‐based SARS‐CoV‐2 study and aid in understanding COVID‐19 pathogenesis and facilitating vaccine development and drug discovery.

## AUTHOR CONTRIBUTIONS

Minghui Li: Conceptualization, supervision, data curation, writing review, and editing. Yuhan Yuan, Ting Zou, and Zongkun Hou: Data curation, review, and editing. Liang Jin and Bochu Wang: Conceptualization, supervision, review and editing.

## CONFLICT OF INTEREST STATEMENT

The authors declare no potential conflicts of interest.

## Supporting information


**Appendix S1:** Supporting information.

## Data Availability

The datasets analyzed during the current research are available from the corresponding author on reasonable request.

## References

[1] WHO . Who coronavirus (COVID‐19) dashboard. 2022 Available from: https://covid19.who.int/

[2] Han Y , Yang L , Lacko LA , Chen S . Human organoid models to study SARS‐CoV‐2 infection. Nat Methods. 2022;19:418‐428. 35396481 10.1038/s41592-022-01453-y

[3] Fan Y , Li X , Zhang L , Wan S , Zhang L , Zhou F . SARS‐CoV‐2 omicron variant: recent progress and future perspectives. Signal Transduct Target Ther. 2022;7:141. 35484110 10.1038/s41392-022-00997-x PMC9047469

[4] Thakur V , Ratho RK . Omicron (b.1.1.529): a new SARS‐CoV‐2 variant of concern mounting worldwide fear. J Med Virol. 2022;94:1821‐1824. 34936120 10.1002/jmv.27541

[5] Chen J , Wang R , Gilby NB , Wei GW . Omicron variant (b.1.1.529): infectivity, vaccine breakthrough, and antibody resistance. J Chem Inf Model. 2022;62:412‐422. 34989238 10.1021/acs.jcim.1c01451 PMC8751645

[6] Cui Z , Liu P , Wang N , et al. Structural and functional characterizations of infectivity and immune evasion of SARS‐CoV‐2 omicron. Cell. 2022;185:860‐871.e13. 35120603 10.1016/j.cell.2022.01.019 PMC8786603

[7] Ai J , Zhang H , Zhang Y , et al. Omicron variant showed lower neutralizing sensitivity than other SARS‐CoV‐2 variants to immune sera elicited by vaccines after boost. Emerg Microbes Infect. 2022;11:337‐343. 34935594 10.1080/22221751.2021.2022440 PMC8788341

[8] Perez‐Then E , Lucas C , Monteiro VS , et al. Neutralizing antibodies against the SARS‐CoV‐2 delta and omicron variants following heterologous coronavac plus bnt162b2 booster vaccination. Nat Med. 2022;28:481‐485. 35051990 10.1038/s41591-022-01705-6 PMC8938264

[9] Yu X , Wei D , Xu W , et al. Reduced sensitivity of SARS‐CoV‐2 omicron variant to antibody neutralization elicited by booster vaccination. Cell Discov. 2022;8:4. 35034952 10.1038/s41421-022-00375-5 PMC8761745

[10] Takayama K . In vitro and animal models for SARS‐CoV‐2 research. Trends Pharmacol Sci. 2020;41:513‐517. 32553545 10.1016/j.tips.2020.05.005 PMC7260555

[11] Yang L , Han Y , Nilsson‐Payant BE , et al. A human pluripotent stem cell‐based platform to study SARS‐CoV‐2 tropism and model virus infection in human cells and organoids. Cell Stem Cell. 2020;27:125‐136.e7. 32579880 10.1016/j.stem.2020.06.015 PMC7303620

[12] Hu J , Li C , Wang S , Li T , Zhang H . Genetic variants are identified to increase risk of COVID‐19 related mortality from UK biobank data. Hum Genomics. 2021;15:10. 33536081 10.1186/s40246-021-00306-7 PMC7856608

[13] Pairo‐Castineira E , Clohisey S , Klaric L , et al. Genetic mechanisms of critical illness in COVID‐19. Nature. 2021;591:92‐98. 33307546 10.1038/s41586-020-03065-y

[14] Drost J , Clevers H . Translational applications of adult stem cell‐derived organoids. Development. 2017;144:968‐975. 28292843 10.1242/dev.140566

[15] Kim J , Koo BK , Knoblich JA . Human organoids: model systems for human biology and medicine. Nat Rev Mol Cell Biol. 2020;21:571‐584. 32636524 10.1038/s41580-020-0259-3 PMC7339799

[16] Rookmaaker MB , Schutgens F , Verhaar MC , Clevers H . Development and application of human adult stem or progenitor cell organoids. Nat Rev Nephrol. 2015;11:546‐554. 26215513 10.1038/nrneph.2015.118

[17] Szabo L , Seubert AC , Kretzschmar K . Modelling adult stem cells and their niche in health and disease with epithelial organoids. Semin Cell Dev Biol. 2023;144:20‐30. 36127261 10.1016/j.semcdb.2022.09.006

[18] Clevers H . Modeling development and disease with organoids. Cell. 2016;165:1586‐1597. 27315476 10.1016/j.cell.2016.05.082

[19] Schutgens F , Rookmaaker MB , Margaritis T , et al. Tubuloids derived from human adult kidney and urine for personalized disease modeling. Nat Biotechnol. 2019;37:303‐313. 30833775 10.1038/s41587-019-0048-8

[20] Schutgens F , Clevers H . Human organoids: tools for understanding biology and treating diseases. Annu Rev Pathol. 2020;15:211‐234. 31550983 10.1146/annurev-pathmechdis-012419-032611

[21] Eiraku M , Takata N , Ishibashi H , et al. Self‐organizing optic‐cup morphogenesis in three‐dimensional culture. Nature. 2011;472:51‐56. 21475194 10.1038/nature09941

[22] Lancaster MA , Renner M , Martin CA , et al. Cerebral organoids model human brain development and microcephaly. Nature. 2013;501:373‐379. 23995685 10.1038/nature12517 PMC3817409

[23] Wimmer RA , Leopoldi A , Aichinger M , et al. Human blood vessel organoids as a model of diabetic vasculopathy. Nature. 2019;565:505‐510. 30651639 10.1038/s41586-018-0858-8 PMC7116578

[24] Li M , Gong J , Gao L , Zou T , Kang J , Xu H . Advanced human developmental toxicity and teratogenicity assessment using human organoid models. Ecotoxicol Environ Saf. 2022;235:113429. 35325609 10.1016/j.ecoenv.2022.113429

[25] Qian X , Nguyen HN , Song MM , et al. Brain‐region‐specific organoids using mini‐bioreactors for modeling zikv exposure. Cell. 2016;165:1238‐1254. 27118425 10.1016/j.cell.2016.04.032 PMC4900885

[26] Gabriel E , Ramani A , Karow U , et al. Recent zika virus isolates induce premature differentiation of neural progenitors in human brain organoids. Cell Stem Cell. 2017;20:397‐406.e5. 28132835 10.1016/j.stem.2016.12.005

[27] Priyathilaka TT , Laaker CJ , Herbath M , Fabry Z , Sandor M . Modeling infectious diseases of the central nervous system with human brain organoids. Transl Res. 2022;250:18‐35. 35811019 10.1016/j.trsl.2022.06.013 PMC11185418

[28] Xu YP , Qiu Y , Zhang B , et al. Zika virus infection induces RNAi‐mediated antiviral immunity in human neural progenitors and brain organoids. Cell Res. 2019;29:265‐273. 30814679 10.1038/s41422-019-0152-9 PMC6461993

[29] Xu M , Lee EM , Wen Z , et al. Identification of small‐molecule inhibitors of zika virus infection and induced neural cell death via a drug repurposing screen. Nat Med. 2016;22:1101‐1107. 27571349 10.1038/nm.4184 PMC5386783

[30] Li C , Deng YQ , Wang S , et al. 25‐hydroxycholesterol protects host against zika virus infection and its associated microcephaly in a mouse model. Immunity. 2017;46:446‐456. 28314593 10.1016/j.immuni.2017.02.012 PMC5957489

[31] Chen C , Song M . Visualizing a field of research: a methodology of systematic scientometric reviews. PLoS One. 2019;14:e0223994. 31671124 10.1371/journal.pone.0223994 PMC6822756

[32] Chen Y , Zhang X , Chen S , et al. Bibliometric analysis of mental health during the COVID‐19 pandemic. Asian J Psychiatr. 2021;65:102846. 34562753 10.1016/j.ajp.2021.102846 PMC8435062

[33] Gu C , Wang Z , Pan Y , Zhu S , Gu Z . Tungsten‐based nanomaterials in the biomedical field: a bibliometric analysis of research progress and prospects. Adv Mater. 2022;35:2204397. 10.1002/adma.202204397 35906814

[34] Li C , Zhu X , Zhang Y , Zhang J , Jeon CO , Jia B . COVID‐19 influences both physical and mental health: lessons from bibliometric analysis. Travel Med Infect Dis. 2022;49:102405. 35917914 10.1016/j.tmaid.2022.102405 PMC9338833

[35] Bornmann L , Daniel HD . The state of h index research. Is the h index the ideal way to measure research performance? EMBO Rep. 2009;10:2‐6. 19079129 10.1038/embor.2008.233 PMC2613214

[36] Hirsch JE . Does the *h* index have predictive power? Proc Natl Acad Sci U S A. 2007;104:19193‐19198. 18040045 10.1073/pnas.0707962104 PMC2148266

[37] Boyack KW , Klavans R . Co‐citation analysis, bibliographic coupling, and direct citation: which citation approach represents the research front most accurately? J Am Soc Inform Sci Technol. 2010;61:2389‐2404.

[38] Trujillo CM , Long TM . Document co‐citation analysis to enhance transdisciplinary research. Sci Adv. 2018;4:e1701130. 29308433 10.1126/sciadv.1701130 PMC5752411

[39] Hoffmann M , Kleine‐Weber H , Schroeder S , et al. SARS‐CoV‐2 cell entry depends on ace2 and tmprss2 and is blocked by a clinically proven protease inhibitor. Cell. 2020;181:271‐280.e8. 32142651 10.1016/j.cell.2020.02.052 PMC7102627

[40] Han Y , Duan X , Yang L , et al. Identification of SARS‐CoV‐2 inhibitors using lung and colonic organoids. Nature. 2021;589:270‐275. 33116299 10.1038/s41586-020-2901-9 PMC8034380

[41] Lamers MM , Beumer J , van der Vaart J , et al. SARS‐CoV‐2 productively infects human gut enterocytes. Science. 2020;369:50‐54. 32358202 10.1126/science.abc1669 PMC7199907

[42] Monteil V , Kwon H , Prado P , et al. Inhibition of SARS‐CoV‐2 infections in engineered human tissues using clinical‐grade soluble human ace2. Cell. 2020;181:905‐913.e7. 32333836 10.1016/j.cell.2020.04.004 PMC7181998

[43] Basil MC , Katzen J , Engler AE , et al. The cellular and physiological basis for lung repair and regeneration: past, present, and future. Cell Stem Cell. 2020;26:482‐502. 32243808 10.1016/j.stem.2020.03.009 PMC7128675

[44] Upadhya S , Rehman J , Malik AB , Chen S . Mechanisms of lung injury induced by SARS‐CoV‐2 infection. Physiology (Bethesda). 2022;37:88‐100. 34698589 10.1152/physiol.00033.2021 PMC8873036

[45] Ziegler CGK , Allon SJ , Nyquist SK , et al. SARS‐CoV‐2 receptor ace2 is an interferon‐stimulated gene in human airway epithelial cells and is detected in specific cell subsets across tissues. Cell. 2020;181:1016‐1035. 32413319 10.1016/j.cell.2020.04.035 PMC7252096

[46] Pei R , Feng J , Zhang Y , et al. Host metabolism dysregulation and cell tropism identification in human airway and alveolar organoids upon SARS‐CoV‐2 infection. Protein Cell. 2021;12:717‐733. 33314005 10.1007/s13238-020-00811-w PMC7732737

[47] Salahudeen AA , Choi SS , Rustagi A , et al. Progenitor identification and SARS‐CoV‐2 infection in human distal lung organoids. Nature. 2020;588:670‐675. 33238290 10.1038/s41586-020-3014-1 PMC8003326

[48] Sano E , Suzuki T , Hashimoto R , et al. Cell response analysis in SARS‐CoV‐2 infected bronchial organoids. Commun Biol. 2022;5:516. 35637255 10.1038/s42003-022-03499-2 PMC9151746

[49] Jacob F , Pather SR , Huang WK , et al. Human pluripotent stem cell‐derived neural cells and brain organoids reveal SARS‐CoV‐2 neurotropism predominates in choroid plexus epithelium. Cell Stem Cell. 2020;27:937‐950.e9. 33010822 10.1016/j.stem.2020.09.016 PMC7505550

[50] Chen KG , Park K , Spence JR . Studying SARS‐CoV‐2 infectivity and therapeutic responses with complex organoids. Nat Cell Biol. 2021;23:822‐833. 34341531 10.1038/s41556-021-00721-x PMC8355201

[51] Chiu MC , Li C , Liu X , et al. Human nasal organoids model SARS‐CoV‐2 upper respiratory infection and recapitulate the differential infectivity of emerging variants. MBio. 2022;13:e0194422. 35938726 10.1128/mbio.01944-22 PMC9426414

[52] Deng X , Garcia‐Knight MA , Khalid MM , et al. Transmission, infectivity, and neutralization of a spike l452r SARS‐CoV‐2 variant. Cell. 2021;184:3426‐3437. 33991487 10.1016/j.cell.2021.04.025 PMC8057738

[53] Honzke K , Obermayer B , Mache C , et al. Human lungs show limited permissiveness for SARS‐CoV‐2 due to scarce ace2 levels but virus‐induced expansion of inflammatory macrophages. Eur Respir J. 2022;60:2102725. 35728978 10.1183/13993003.02725-2021 PMC9712848

[54] Lamers MM , van der Vaart J , Knoops K , et al. An organoid‐derived bronchioalveolar model for SARS‐CoV‐2 infection of human alveolar type ii‐like cells. EMBO J. 2021;40:e105912. 33283287 10.15252/embj.2020105912 PMC7883112

[55] Mlcochova P , Kemp SA , Dhar MS , et al. SARS‐CoV‐2 b.1.617.2 delta variant replication and immune evasion. Nature. 2021;599:114‐119. 34488225 10.1038/s41586-021-03944-y PMC8566220

[56] Ahn JH , Kim J , Hong SP , et al. Nasal ciliated cells are primary targets for SARS‐CoV‐2 replication in the early stage of COVID‐19. J Clin Invest. 2021;131:e148517. 34003804 10.1172/JCI148517 PMC8245175

[57] Wu CT , Lidsky PV , Xiao Y , et al. SARS‐CoV‐2 replication in airway epithelia requires motile cilia and microvillar reprogramming. Cell. 2023;186:112‐130.e20. 36580912 10.1016/j.cell.2022.11.030 PMC9715480

[58] Elbadawi M , Efferth T . Organoids of human airways to study infectivity and cytopathy of SARS‐CoV‐2. Lancet Respir Med. 2020;8:e55‐e56. 32446313 10.1016/S2213-2600(20)30238-1 PMC7241998

[59] Sachs N , Papaspyropoulos A , Zomer‐van Ommen DD , et al. Long‐term expanding human airway organoids for disease modeling. EMBO J. 2019;38:e100300. 30643021 10.15252/embj.2018100300 PMC6376275

[60] Duan X , Tang X , Nair MS , et al. An airway organoid‐based screen identifies a role for the hif1alpha‐glycolysis axis in SARS‐CoV‐2 infection. Cell Rep. 2021;37:109920. 34731648 10.1016/j.celrep.2021.109920 PMC8516798

[61] Blanco‐Melo D , Nilsson‐Payant BE , Liu WC , et al. Imbalanced host response to SARS‐CoV‐2 drives development of COVID‐19. Cell. 2020;181:1036‐1045.e9. 32416070 10.1016/j.cell.2020.04.026 PMC7227586

[62] Suzuki T , Itoh Y , Sakai Y , et al. Generation of human bronchial organoids for SARS‐CoV‐2 research. bioRxiv. 2020.

[63] Chen F , Fine A . Stem cells in lung injury and repair. Am J Pathol. 2016;186:2544‐2550. 27524796 10.1016/j.ajpath.2016.05.023 PMC5222968

[64] Fang KY , Cao WC , Xie TA , et al. Exploration and validation of related hub gene expression during SARS‐CoV‐2 infection of human bronchial organoids. Hum Genomics. 2021;15:18. 33726831 10.1186/s40246-021-00316-5 PMC7962432

[65] Chiu MC , Li C , Liu X , et al. A bipotential organoid model of respiratory epithelium recapitulates high infectivity of SARS‐CoV‐2 omicron variant. Cell Discov. 2022;8:57. 35710786 10.1038/s41421-022-00422-1 PMC9203776

[66] Tiwari SK , Wang S , Smith D , Carlin AF , Rana TM . Revealing tissue‐specific SARS‐CoV‐2 infection and host responses using human stem cell‐derived lung and cerebral organoids. Stem Cell Rep. 2021;16:437‐445. 10.1016/j.stemcr.2021.02.005 PMC7879814 33631122

[67] Katsura H , Sontake V , Tata A , et al. Human lung stem cell‐based alveolospheres provide insights into SARS‐CoV‐2‐mediated interferon responses and pneumocyte dysfunction. Cell Stem Cell. 2020;27:890‐904.e898. 33128895 10.1016/j.stem.2020.10.005 PMC7577733

[68] Youk J , Kim T , Evans KV , et al. Three‐dimensional human alveolar stem cell culture models reveal infection response to SARS‐CoV‐2. Cell Stem Cell. 2020;27:905‐919.e10. 33142113 10.1016/j.stem.2020.10.004 PMC7577700

[69] Chan M . Hkumed finds omicron SARS‐CoV‐2 can infect faster and better than delta in human bronchus but with less severe infection in lung. Braz J Implantol Health Sci. 2022;4:50‐54.

[70] Ellul MA , Benjamin L , Singh B , et al. Neurological associations of COVID‐19. Lancet Neurol. 2020;19:767‐783. 32622375 10.1016/S1474-4422(20)30221-0 PMC7332267

[71] Iadecola C , Anrather J , Kamel H . Effects of COVID‐19 on the nervous system. Cell. 2020;183:16‐27.e1. 32882182 10.1016/j.cell.2020.08.028 PMC7437501

[72] Kral AH , Lambdin BH , Wenger LD , Davidson PJ . Evaluation of an unsanctioned safe consumption site in the United States. N Engl J Med. 2020;383:589‐590. 32640126 10.1056/NEJMc2015435

[73] Vanderver A , Adang L , Gavazzi F , et al. Janus kinase inhibition in the aicardi‐goutieres syndrome. N Engl J Med. 2020;383:986‐989. 32877590 10.1056/NEJMc2001362 PMC7495410

[74] Zhan M , Qin Y , Xue X , Zhu S . Death from COVID‐19 of 23 health care workers in China. N Engl J Med. 2020;382:2267‐2268. 32294342 10.1056/NEJMc2005696 PMC7179960

[75] Ramani A , Muller L , Ostermann PN , et al. SARS‐CoV‐2 targets neurons of 3d human brain organoids. EMBO J. 2020;39:e106230. 32876341 10.15252/embj.2020106230 PMC7560208

[76] Song E , Zhang C , Israelow B , et al. Neuroinvasion of SARS‐CoV‐2 in human and mouse brain. J Exp Med. 2021;218:e20202135. 33433624 10.1084/jem.20202135 PMC7808299

[77] Cugola FR , Fernandes IR , Russo FB , et al. The brazilian zika virus strain causes birth defects in experimental models. Nature. 2016;534:267‐271. 27279226 10.1038/nature18296 PMC4902174

[78] Hou Y , Li C , Yoon C , et al. Enhanced replication of SARS‐CoV‐2 omicron ba.2 in human forebrain and midbrain organoids. Signal Transduct Target Ther. 2022;7:381. 36411276 10.1038/s41392-022-01241-2 PMC9676899

[79] Pellegrini L , Albecka A , Mallery DL , et al. SARS‐CoV‐2 infects the brain choroid plexus and disrupts the blood‐csf barrier in human brain organoids. Cell Stem Cell. 2020;27:951‐961.e5. 33113348 10.1016/j.stem.2020.10.001 PMC7553118

[80] Chen R , Wang K , Yu J , et al. The spatial and cell‐type distribution of SARS‐CoV‐2 receptor ace2 in the human and mouse brains. Front Neurol. 2020;11:573095. 33551947 10.3389/fneur.2020.573095 PMC7855591

[81] Zhang BZ , Chu H , Han S , et al. SARS‐CoV‐2 infects human neural progenitor cells and brain organoids. Cell Res. 2020;30:928‐931. 32753756 10.1038/s41422-020-0390-x PMC7399356

[82] Wang C , Zhang M , Garcia G Jr , et al. Apoe‐isoform‐dependent SARS‐CoV‐2 neurotropism and cellular response. Cell Stem Cell. 2021;28:331‐342.e5. 33450186 10.1016/j.stem.2020.12.018 PMC7832490

[83] Ferm S , Fisher C , Pakala T , et al. Analysis of gastrointestinal and hepatic manifestations of SARS‐CoV‐2 infection in 892 patients in queens. Clin Gastroenterol Hepatol. 2020;18:2378‐2379.e2371. 32497637 10.1016/j.cgh.2020.05.049 PMC7263206

[84] Guo M , Tao W , Flavell RA , Zhu S . Potential intestinal infection and faecal‐oral transmission of SARS‐CoV‐2. Nat Rev Gastroenterol Hepatol. 2021;18:269‐283. 33589829 10.1038/s41575-021-00416-6 PMC7883337

[85] Pan L , Mu M , Yang P , et al. Clinical characteristics of COVID‐19 patients with digestive symptoms in Hubei, China: a descriptive, cross‐sectional, multicenter study. Am J Gastroenterol. 2020;115:766‐773. 32287140 10.14309/ajg.0000000000000620 PMC7172492

[86] Wang W , Xu Y , Gao R , et al. Detection of SARS‐CoV‐2 in different types of clinical specimens. JAMA. 2020;323:1843‐1844. 32159775 10.1001/jama.2020.3786 PMC7066521

[87] Xiao F , Tang M , Zheng X , Liu Y , Li X , Shan H . Evidence for gastrointestinal infection of SARS‐CoV‐2. Gastroenterology. 2020;158:1831‐1833.e3. 32142773 10.1053/j.gastro.2020.02.055 PMC7130181

[88] Kruger J , Gross R , Conzelmann C , et al. Drug inhibition of SARS‐CoV‐2 replication in human pluripotent stem cell‐derived intestinal organoids. Cell Mol Gastroenterol Hepatol. 2021;11:935‐948. 33186749 10.1016/j.jcmgh.2020.11.003 PMC7655023

[89] Zhao Y , Ren X , Lu J , et al. Mechanistic insight of SARS‐CoV‐2 infection using human hepatobiliary organoids. Gut. 2022;72:216‐218. 35459707 10.1136/gutjnl-2021-326617 PMC9763169

[90] Garreta E , Prado P , Stanifer ML , et al. A diabetic milieu increases ace2 expression and cellular susceptibility to SARS‐CoV‐2 infections in human kidney organoids and patient cells. Cell Metab. 2022;34:857‐873.e9. 35561674 10.1016/j.cmet.2022.04.009 PMC9097013

[91] Menuchin‐Lasowski Y , Schreiber A , Lecanda A , et al. SARS‐CoV‐2 infects and replicates in photoreceptor and retinal ganglion cells of human retinal organoids. Stem Cell Rep. 2022;17:789‐803. 10.1016/j.stemcr.2022.02.015 PMC8943915 35334213

[92] Tanaka J , Senpuku H , Ogawa M , et al. Human induced pluripotent stem cell‐derived salivary gland organoids model SARS‐CoV‐2 infection and replication. Nat Cell Biol. 2022;24:1595‐1605. 36253535 10.1038/s41556-022-01007-6 PMC11580836

[93] Giobbe GG , Bonfante F , Jones BC , et al. SARS‐CoV‐2 infection and replication in human gastric organoids. Nat Commun. 2021;12:6610. 34785679 10.1038/s41467-021-26762-2 PMC8595698

[94] Jang KK , Kaczmarek ME , Dallari S , et al. Variable susceptibility of intestinal organoid‐derived monolayers to SARS‐CoV‐2 infection. PLoS Biol. 2022;20:e3001592. 35358182 10.1371/journal.pbio.3001592 PMC9004766

[95] Wang Y , Zhang D , Du G , et al. Remdesivir in adults with severe COVID‐19: a randomised, double‐blind, placebo‐controlled, multicentre trial. Lancet. 2020;395:1569‐1578. 32423584 10.1016/S0140-6736(20)31022-9 PMC7190303

[96] Zhou J , Li C , Liu X , et al. Infection of bat and human intestinal organoids by SARS‐CoV‐2. Nat Med. 2020;26:1077‐1083. 32405028 10.1038/s41591-020-0912-6

[97] Zang R , Gomez Castro MF , McCune BT , et al. Tmprss2 and tmprss4 promote SARS‐CoV‐2 infection of human small intestinal enterocytes. Sci Immunol. 2020;5:eabc3582. 32404436 10.1126/sciimmunol.abc3582 PMC7285829

[98] Heuberger J , Trimpert J , Vladimirova D , et al. Epithelial response to IFN‐gamma promotes SARS‐CoV‐2 infection. EMBO Mol Med. 2021;13:e13191. 33544398 10.15252/emmm.202013191 PMC7995094

[99] Zhao Z , Chen X , Dowbaj AM , et al. Organoids. Nat Rev Methods Prim. 2022;2:94. doi:10.1038/s43586-022-00174-y. PMC10270325 37325195

[100] Triana S , Metz‐Zumaran C , Ramirez C , et al. Single‐cell analyses reveal SARS‐CoV‐2 interference with intrinsic immune response in the human gut. Mol Syst Biol. 2021;17:e10232. 33904651 10.15252/msb.202110232 PMC8077299

[101] Fan Z , Chen L , Li J , et al. Clinical features of COVID‐19‐related liver functional abnormality. Clin Gastroenterol Hepatol. 2020;18:1561‐1566. 32283325 10.1016/j.cgh.2020.04.002 PMC7194865

[102] Huang C , Wang Y , Li X , et al. Clinical features of patients infected with 2019 novel coronavirus in Wuhan, China. Lancet. 2020;395:497‐506. 31986264 10.1016/S0140-6736(20)30183-5 PMC7159299

[103] Richards A , Friesen M , Khalil A , Barrasa MI , Gehrke L , Jaenisch R . SARS‐CoV‐2 infection of human pluripotent stem cell‐derived liver organoids reveals potential mechanisms of liver pathology. iScience. 2022;25:105146. 36128218 10.1016/j.isci.2022.105146 PMC9477603

[104] Zhao B , Ni C , Gao R , et al. Recapitulation of SARS‐CoV‐2 infection and cholangiocyte damage with human liver ductal organoids. Protein Cell. 2020;11:771‐775. 32303993 10.1007/s13238-020-00718-6 PMC7164704

[105] Soler MJ , Jacobs‐Cacha C . The COVID‐19 pandemic: progress in nephrology. Nat Rev Nephrol. 2022;18:80‐81. 34873314 10.1038/s41581-021-00521-4 PMC8646337

[106] Guan WJ , Ni ZY , Hu Y , et al. Clinical characteristics of coronavirus disease 2019 in China. N Engl J Med. 2020;382:1708‐1720. 32109013 10.1056/NEJMoa2002032 PMC7092819

[107] Li Z , Wu M , Yao J , Guo J , Liao X , Song S , et al. Caution on kidney dysfunctions of COVID‐19 patients. medRXiv. 2020.

[108] Pan XW , Xu D , Zhang H , Zhou W , Wang LH , Cui XG . Identification of a potential mechanism of acute kidney injury during the COVID‐19 outbreak: a study based on single‐cell transcriptome analysis. Intensive Care Med. 2020;46:1114‐1116. 32236644 10.1007/s00134-020-06026-1 PMC7106051

[109] Xia S , Wu M , Chen S , et al. Long term culture of human kidney proximal tubule epithelial cells maintains lineage functions and serves as an ex vivo model for coronavirus associated kidney injury. Virol Sin. 2020;35:311‐320. 32602046 10.1007/s12250-020-00253-y PMC7322379

[110] Jansen J , Reimer KC , Nagai JS , et al. SARS‐CoV‐2 infects the human kidney and drives fibrosis in kidney organoids. Cell Stem Cell. 2022;29:217‐231. 35032430 10.1016/j.stem.2021.12.010 PMC8709832

[111] Guo T , Fan Y , Chen M , et al. Cardiovascular implications of fatal outcomes of patients with coronavirus disease 2019 (COVID‐19). JAMA Cardiol. 2020;5:811‐818. 32219356 10.1001/jamacardio.2020.1017 PMC7101506

[112] Shi S , Qin M , Shen B , et al. Association of cardiac injury with mortality in hospitalized patients with COVID‐19 in Wuhan, China. JAMA Cardiol. 2020;5:802‐810. 32211816 10.1001/jamacardio.2020.0950 PMC7097841

[113] Arhontoulis DC , Kerr CM , Richards D , et al. Human cardiac organoids to model COVID‐19 cytokine storm induced cardiac injuries. J Tissue Eng Regen Med. 2022;16:799‐811. 35689600 10.1002/term.3327 PMC9350263

[114] Khan AO , Reyat JS , Hill H , et al. Preferential uptake of SARS‐CoV‐2 by pericytes potentiates vascular damage and permeability in an organoid model of the microvasculature. Cardiovasc Res. 2022;118:3085‐3096. 35709328 10.1093/cvr/cvac097 PMC9214165

[115] Mills RJ , Humphrey SJ , Fortuna PRJ , et al. Bet inhibition blocks inflammation‐induced cardiac dysfunction and SARS‐CoV‐2 infection. Cell. 2021;184:2167‐2182.e22. 33811809 10.1016/j.cell.2021.03.026 PMC7962543

[116] Eriksen AZ , Moller R , Makovoz B , Uhl SA , tenOever BR , Blenkinsop TA . SARS‐CoV‐2 infects human adult donor eyes and hesc‐derived ocular epithelium. Cell Stem Cell. 2021;28:1205‐1220.e7. 34022129 10.1016/j.stem.2021.04.028 PMC8126605

[117] Ahmad Mulyadi Lai HI , Chou SJ , Chien Y , et al. Expression of endogenous angiotensin‐converting enzyme 2 in human induced pluripotent stem cell‐derived retinal organoids. Int J Mol Sci. 2021;22:1320. 33525682 10.3390/ijms22031320 PMC7865454

[118] Xu J , Li Y , Gan F , Du Y , Yao Y . Salivary glands: potential reservoirs for COVID‐19 asymptomatic infection. J Dent Res. 2020;99:989. 32271653 10.1177/0022034520918518

[119] Li Y , Tenchov R , Smoot J , Liu C , Watkins S , Zhou Q . A comprehensive review of the global efforts on COVID‐19 vaccine development. ACS Cent Sci. 2021;7:512‐533. 34056083 10.1021/acscentsci.1c00120 PMC8029445

[120] Dong Y , Dai T , Wei Y , Zhang L , Zheng M , Zhou F . A systematic review of SARS‐CoV‐2 vaccine candidates. Signal Transduct Target Ther. 2020;5:237. 33051445 10.1038/s41392-020-00352-y PMC7551521

[121] Krammer F . SARS‐CoV‐2 vaccines in development. Nature. 2020;586:516‐527. 32967006 10.1038/s41586-020-2798-3

[122] Thanh Le T , Andreadakis Z , Kumar A , et al. The COVID‐19 vaccine development landscape. Nat Rev Drug Discov. 2020;19:305‐306. 32273591 10.1038/d41573-020-00073-5

[123] WHO . COVID‐19 vaccine tracker and landscape. 2022 Available from: https://www.who.int/publications/m/item/draft-landscape-of-covid-19-candidate-vaccines

[124] Busquet F , Hartung T , Pallocca G , Rovida C , Leist M . Harnessing the power of novel animal‐free test methods for the development of COVID‐19 drugs and vaccines. Arch Toxicol. 2020;94:2263‐2272. 32447523 10.1007/s00204-020-02787-2 PMC7245508

[125] Herati RS , Wherry EJ . What is the predictive value of animal models for vaccine efficacy in humans? Consideration of strategies to improve the value of animal models. Cold Spring Harb Perspect Biol. 2018;10:a031583. 28348037 10.1101/cshperspect.a031583 PMC5880169

[126] Wagar LE , Salahudeen A , Constantz CM , et al. Modeling human adaptive immune responses with tonsil organoids. Nat Med. 2021;27:125‐135. 33432170 10.1038/s41591-020-01145-0 PMC7891554

[127] Watkins DI , Burton DR , Kallas EG , Moore JP , Koff WC . Nonhuman primate models and the failure of the merck HIV‐1 vaccine in humans. Nat Med. 2008;14:617‐621. 18535579 10.1038/nm.f.1759 PMC3697853

[128] Shi R , Shan C , Duan X , et al. A human neutralizing antibody targets the receptor‐binding site of SARS‐CoV‐2. Nature. 2020;584:120‐124. 32454512 10.1038/s41586-020-2381-y

[129] Spitalieri P , Centofanti F , Murdocca M , et al. Two different therapeutic approaches for SARS‐CoV‐2 in hiPSCs‐derived lung organoids. Cells. 2022;11:1235. 35406799 10.3390/cells11071235 PMC8997767

[130] Giese C , Marx U . Human immunity in vitro—solving immunogenicity and more. Adv Drug Deliv Rev. 2014;69‐70:103‐122. 10.1016/j.addr.2013.12.011 24447895

[131] Goyal G , Prabhala P , Mahajan G , et al. Ectopic lymphoid follicle formation and human seasonal influenza vaccination responses recapitulated in an organ‐on‐a‐chip. Adv Sci (Weinh). 2022;9:e2103241. 35289122 10.1002/advs.202103241 PMC9109055

[132] Hammel JH , Zatorski JM , Cook SR , Pompano RR , Munson JM . Engineering in vitro immune‐competent tissue models for testing and evaluation of therapeutics. Adv Drug Deliv Rev. 2022;182:114111. 35031388 10.1016/j.addr.2022.114111 PMC8908413

[133] Mesci P , de Souza JS , Martin‐Sancho L , et al. SARS‐CoV‐2 infects human brain organoids causing cell death and loss of synapses that can be rescued by treatment with sofosbuvir. PLoS Biol. 2022;20:e3001845. 36327326 10.1371/journal.pbio.3001845 PMC9632769

[134] Wagar L . Small centers of defense. Science. 2022;375:830. 35201866 10.1126/science.abn9652

[135] Duarte RRR , Copertino DC Jr , Iniguez LP , et al. Identifying fda‐approved drugs with multimodal properties against COVID‐19 using a data‐driven approach and a lung organoid model of SARS‐CoV‐2 entry. Mol Med. 2021;27:105. 34503440 10.1186/s10020-021-00356-6 PMC8426591

[136] Peng L , Gao L , Wu X , et al. Lung organoids as model to study SARS‐CoV‐2 infection. Cells. 2022;11:2758. 10.3390/cells11172758 PMC9455466 36078166

[137] Xu G , Li Y , Zhang S , et al. SARS‐CoV‐2 promotes ripk1 activation to facilitate viral propagation. Cell Res. 2021;31:1230‐1243. 34663909 10.1038/s41422-021-00578-7 PMC8522117

[138] Huang KY , Lin MS , Kuo TC , et al. Humanized COVID‐19 decoy antibody effectively blocks viral entry and prevents SARS‐CoV‐2 infection. EMBO Mol Med. 2021;13:e12828. 33159417 10.15252/emmm.202012828 PMC7799362

[139] Samuel RM , Majd H , Richter MN , et al. Androgen signaling regulates SARS‐CoV‐2 receptor levels and is associated with severe COVID‐19 symptoms in men. Cell Stem Cell. 2020;27:876‐889.e12. 33232663 10.1016/j.stem.2020.11.009 PMC7670929

[140] Mulay A , Konda B , Garcia G Jr , et al. SARS‐CoV‐2 infection of primary human lung epithelium for COVID‐19 modeling and drug discovery. Cell Rep. 2021;35:109055. 33905739 10.1016/j.celrep.2021.109055 PMC8043574

[141] Beigel JH , Tomashek KM , Dodd LE , et al. Remdesivir for the treatment of COVID‐19—final report. N Engl J Med. 2020;383:1813‐1826. 32445440 10.1056/NEJMoa2007764 PMC7262788

[142] Zhang M , Wang P , Luo R , et al. Biomimetic human disease model of SARS‐CoV‐2‐induced lung injury and immune responses on organ chip system. Adv Sci (Weinh). 2021;8:2002928. 33173719 10.1002/advs.202002928 PMC7646023

[143] Stanifer ML , Kee C , Cortese M , et al. Critical role of type iii interferon in controlling SARS‐CoV‐2 infection in human intestinal epithelial cells. Cell Rep. 2020;32:107863. 32610043 10.1016/j.celrep.2020.107863 PMC7303637

[144] Andreakos E , Tsiodras S . COVID‐19: lambda interferon against viral load and hyperinflammation. EMBO Mol Med. 2020;12:e12465. 32333818 10.15252/emmm.202012465 PMC7267110

[145] Jhuti D , Rawat A , Guo CM , Wilson LA , Mills EJ , Forrest JI . Interferon treatments for SARS‐CoV‐2: challenges and opportunities. Infect Dis Ther. 2022;11:953‐972. 35445964 10.1007/s40121-022-00633-9 PMC9022612

[146] Si L , Bai H , Rodas M , et al. A human‐airway‐on‐a‐chip for the rapid identification of candidate antiviral therapeutics and prophylactics. Nat Biomed Eng. 2021;5:815‐829. 33941899 10.1038/s41551-021-00718-9 PMC8387338

[147] Samudyata OAO , Malwade S , Rufino de Sousa N , Goparaju SK , Gracias J , et al. SARS‐CoV‐2 promotes microglial synapse elimination in human brain organoids. Mol Psychiatry. 2022;27:3939‐3950. 36198765 10.1038/s41380-022-01786-2 PMC9533278

[148] Wang L , Sievert D , Clark AE , et al. A human three‐dimensional neural‐perivascular ‘assembloid’ promotes astrocytic development and enables modeling of SARS‐CoV‐2 neuropathology. Nat Med. 2021;27:1600‐1606. 34244682 10.1038/s41591-021-01443-1 PMC8601037

[149] Guo Y , Luo R , Wang Y , et al. SARS‐CoV‐2 induced intestinal responses with a biomimetic human gut‐on‐chip. Sci Bull (Beijing). 2021;66:783‐793. 33282445 10.1016/j.scib.2020.11.015 PMC7704334

[150] Huh D , Hamilton GA , Ingber DE . From 3D cell culture to organs‐on‐chips. Trends Cell Biol. 2011;21:745‐754. 22033488 10.1016/j.tcb.2011.09.005 PMC4386065

[151] Zhang YS , Aleman J , Shin SR , et al. Multisensor‐integrated organs‐on‐chips platform for automated and continual in situ monitoring of organoid behaviors. Proc Natl Acad Sci U S A. 2017;114:E2293‐E2302. 28265064 10.1073/pnas.1612906114 PMC5373350

[152] Xia S , Liu M , Wang C , et al. Inhibition of SARS‐CoV‐2 (previously 2019–nCoV) infection by a highly potent pan‐coronavirus fusion inhibitor targeting its spike protein that harbors a high capacity to mediate membrane fusion. Cell Res. 2020;30:343‐355. 32231345 10.1038/s41422-020-0305-x PMC7104723

[153] Wang S , Li W , Hui H , et al. Cholesterol 25‐hydroxylase inhibits SARS‐CoV‐2 and other coronaviruses by depleting membrane cholesterol. EMBO J. 2020;39:e106057. 32944968 10.15252/embj.2020106057 PMC7537045

[154] Freedberg DE , Conigliaro J , Wang TC , et al. Famotidine use is associated with improved clinical outcomes in hospitalized COVID‐19 patients: a propensity score matched retrospective cohort study. Gastroenterology. 2020;159:1129‐1131.e3. 32446698 10.1053/j.gastro.2020.05.053 PMC7242191

[155] Prelli Bozzo C , Nchioua R , Volcic M , et al. Ifitm proteins promote SARS‐CoV‐2 infection and are targets for virus inhibition in vitro. Nat Commun. 2021;12:4584. 34321474 10.1038/s41467-021-24817-y PMC8319209

[156] Brevini T , Maes M , Webb GJ , et al. Fxr inhibition may protect from SARS‐CoV‐2 infection by reducing ace2. Nature. 2023;615:134‐142. 36470304 10.1038/s41586-022-05594-0 PMC9977684

[157] Wysocki J , Ye M , Hassler L , et al. A novel soluble ace2 variant with prolonged duration of action neutralizes SARS‐CoV‐2 infection in human kidney organoids. J Am Soc Nephrol. 2021;32:795‐803. 33526471 10.1681/ASN.2020101537 PMC8017551

[158] Monteil V , Dyczynski M , Lauschke VM , et al. Human soluble ace2 improves the effect of remdesivir in SARS‐CoV‐2 infection. EMBO Mol Med. 2021;13:e13426. 33179852 10.15252/emmm.202013426 PMC7799356

[159] Calistri A , Luganini A , Mognetti B , et al. The new generation hdhodh inhibitor meds433 hinders the in vitro replication of SARS‐CoV‐2 and other human coronaviruses. Microorganisms. 2021;9:1731. 34442810 10.3390/microorganisms9081731 PMC8398173

[160] Kim HK , Kim H , Lee MK , et al. Generation of tonsil organoids as an ex vivo model for SARS‐CoV‐2 infection. Biomaterials. 2020;283:121460. 10.1016/j.biomaterials.2022.121460 PMC8901203 35286852

[161] Gao CC , Li M , Deng W , et al. Differential transcriptomic landscapes of multiple organs from SARS‐CoV‐2 early infected rhesus macaques. Protein Cell. 2022;13:920‐939. 35377064 10.1007/s13238-022-00915-5 PMC8978510

[162] Liu J , Li Y , Liu Q , et al. SARS‐CoV‐2 cell tropism and multiorgan infection. Cell Discov. 2021;7:17. 33758165 10.1038/s41421-021-00249-2 PMC7987126

[163] Vabret N , Britton GJ , Gruber C , et al. Immunology of COVID‐19: current state of the science. Immunity. 2020;52:910‐941. 32505227 10.1016/j.immuni.2020.05.002 PMC7200337

[164] Yang L , Han Y , Jaffre F , et al. An immuno‐cardiac model for macrophage‐mediated inflammation in COVID‐19 hearts. Circ Res. 2021;129:33‐46. 33853355 10.1161/CIRCRESAHA.121.319060 PMC8225586

[165] Muffat J , Li Y , Omer A , et al. Human induced pluripotent stem cell‐derived glial cells and neural progenitors display divergent responses to zika and dengue infections. Proc Natl Acad Sci U S A. 2018;115:7117‐7122. 29915057 10.1073/pnas.1719266115 PMC6142255

[166] Ormel PR , Vieira de Sa R , van Bodegraven EJ , et al. Microglia innately develop within cerebral organoids. Nat Commun. 2018;9:4167. 30301888 10.1038/s41467-018-06684-2 PMC6177485

[167] Popova G , Soliman SS , Kim CN , et al. Human microglia states are conserved across experimental models and regulate neural stem cell responses in chimeric organoids. Cell Stem Cell. 2021;28:2153‐2166. 34536354 10.1016/j.stem.2021.08.015 PMC8642295

[168] Xu R , Boreland AJ , Li X , et al. Developing human pluripotent stem cell‐based cerebral organoids with a controllable microglia ratio for modeling brain development and pathology. Stem Cell Reports. 2021;16:1923‐1937. 34297942 10.1016/j.stemcr.2021.06.011 PMC8365109

[169] Zhang W , Jiang J , Xu Z , et al. Microglia‐containing human brain organoids for the study of brain development and pathology. Mol Psychiatry. 2022;28:96‐107. 36474001 10.1038/s41380-022-01892-1 PMC9734443

[170] Awogbindin IO , Ben‐Azu B , Olusola BA , et al. Microglial implications in SARS‐CoV‐2 infection and COVID‐19: lessons from viral RNA neurotropism and possible relevance to parkinson's disease. Front Cell Neurosci. 2021;15:670298. 34211370 10.3389/fncel.2021.670298 PMC8240959

[171] Frank MG , Nguyen KH , Ball JB , et al. SARS‐CoV‐2 spike s1 subunit induces neuroinflammatory, microglial and behavioral sickness responses: evidence of pamp‐like properties. Brain Behav Immun. 2022;100:267‐277. 34915155 10.1016/j.bbi.2021.12.007 PMC8667429

[172] Poloni TE , Medici V , Moretti M , et al. COVID‐19‐related neuropathology and microglial activation in elderly with and without dementia. Brain Pathol. 2021;31:e12997. 34145669 10.1111/bpa.12997 PMC8412067

[173] DeFalco T , Bhattacharya I , Williams AV , Sams DM , Capel B . Yolk‐sac‐derived macrophages regulate fetal testis vascularization and morphogenesis. Proc Natl Acad Sci U S A. 2014;111:E2384‐E2393. 24912173 10.1073/pnas.1400057111 PMC4060703

[174] Ewald ML , Chen YH , Lee AP , Hughes CCW . The vascular niche in next generation microphysiological systems. Lab Chip. 2021;21:3244‐3262. 34396383 10.1039/d1lc00530h PMC8635227

[175] Grebenyuk S , Ranga A . Engineering organoid vascularization. Front Bioeng Biotechnol. 2019;7:39. 30941347 10.3389/fbioe.2019.00039 PMC6433749

[176] Li M , Gao L , Zhao L , Zou T , Xu H . Toward the next generation of vascularized human neural organoids. Med Res Rev. 2023;43:31‐54. 35993813 10.1002/med.21922

[177] Vargas‐Valderrama A , Messina A , Mitjavila‐Garcia MT , Guenou H . The endothelium, a key actor in organ development and hpsc‐derived organoid vascularization. J Biomed Sci. 2020;27:67. 32443983 10.1186/s12929-020-00661-y PMC7245026

[178] Bargehr J , Rericha P , Petchey A , et al. Cardiovascular ace2 receptor expression in patients undergoing heart transplantation. ESC Heart Fail. 2021;8:4119‐4129. 34390216 10.1002/ehf2.13528 PMC8497226

[179] Buzhdygan TP , DeOre BJ , Baldwin‐Leclair A , et al. The SARS‐CoV‐2 spike protein alters barrier function in 2d static and 3d microfluidic in‐vitro models of the human blood‐brain barrier. Neurobiol Dis. 2020;146:105131. 33053430 10.1016/j.nbd.2020.105131 PMC7547916

[180] Hashimoto R , Takahashi J , Shirakura K , et al. SARS‐CoV‐2 disrupts respiratory vascular barriers by suppressing claudin‐5 expression. Sci Adv. 2022;8:eabo6783. 36129989 10.1126/sciadv.abo6783 PMC9491726

[181] Lee SH , Sung JH . Organ‐on‐a‐chip technology for reproducing multiorgan physiology. Adv Healthc Mater. 2018;7:1700419. 10.1002/adhm.201700419 28945001

[182] Ching T , Toh YC , Hashimoto M , Zhang YS . Bridging the academia‐to‐industry gap: organ‐on‐a‐chip platforms for safety and toxicology assessment. Trends Pharmacol Sci. 2021;42:715‐728. 34187693 10.1016/j.tips.2021.05.007 PMC8364498

[183] Truskey GA . Human microphysiological systems and organoids as in vitro models for toxicological studies. Front Public Health. 2018;6:185. 30042936 10.3389/fpubh.2018.00185 PMC6048981

[184] Picollet‐D'hahan N , Zuchowska A , Lemeunier I , Le Gac S . Multiorgan‐on‐a‐chip: a systemic approach to model and decipher inter‐organ communication. Trends Biotechnol. 2021;39:788‐810. 33541718 10.1016/j.tibtech.2020.11.014

[185] Ramezankhani R , Solhi R , Chai YC , Vosough M , Verfaillie C . Organoid and microfluidics‐based platforms for drug screening in COVID‐19. Drug Discov Today. 2022;27:1062‐1076. 34954328 10.1016/j.drudis.2021.12.014 PMC8695520

[186] Goncalves IM , Carvalho V , Rodrigues RO , et al. Organ‐on‐a‐chip platforms for drug screening and delivery in tumor cells: a systematic review. Cancers (Basel). 2022;14:935. 35205683 10.3390/cancers14040935 PMC8870045

[187] Clarke GA , Hartse BX , Niaraki Asli AE , et al. Advancement of sensor integrated organ‐on‐chip devices. Sensors (Basel). 2021;21:21. 10.3390/s21041367 PMC7922590 33671996

[188] Lopez‐Muñoz GA , Mughal S , Ramón‐Azcón J . Sensors and biosensors in organs‐on‐a‐chip platforms. Adv Exp Med Biol. 2022;1379:55‐80. 35760988 10.1007/978-3-031-04039-9_3

[189] Bost P , Giladi A , Liu Y , et al. Host–viral infection maps reveal signatures of severe COVID‐19 patients. Cell. 2020;181:1475‐1488.e1412. 32479746 10.1016/j.cell.2020.05.006 PMC7205692

[190] Ho DW , Tsui YM , Chan LK , et al. Single‐cell RNA sequencing shows the immunosuppressive landscape and tumor heterogeneity of hbv‐associated hepatocellular carcinoma. Nat Commun. 2021;12:3684. 34140495 10.1038/s41467-021-24010-1 PMC8211687

[191] Kotliar D , Lin AE , Logue J , et al. Single‐cell profiling of ebola virus disease in vivo reveals viral and host dynamics. Cell. 2020;183:1383‐1401.e1319. 33159858 10.1016/j.cell.2020.10.002 PMC7707107

[192] Gao KM , Derr AG , Guo Z , et al. Human nasal wash RNA‐seq reveals distinct cell‐specific innate immune responses in influenza versus SARS‐CoV‐2. JCI Insight. 2021;6:e152288. 34618691 10.1172/jci.insight.152288 PMC8663782

[193] Steuerman Y , Cohen M , Peshes‐Yaloz N , et al. Dissection of influenza infection in vivo by single‐cell RNA sequencing. Cell Syst. 2018;6:679‐691.e674. 29886109 10.1016/j.cels.2018.05.008 PMC7185763

[194] Patiño M , Lagos WN , Patne NS , Tasic B , Zeng H , Callaway EM . Single‐cell transcriptomic classification of rabies‐infected cortical neurons. Proc Natl Acad Sci U S A. 2022;119:e2203677119. 35609197 10.1073/pnas.2203677119 PMC9295789

[195] Kazer SW , Aicher TP , Muema DM , et al. Integrated single‐cell analysis of multicellular immune dynamics during hyperacute HIV‐1 infection. Nat Med. 2020;26:511‐518. 32251406 10.1038/s41591-020-0799-2 PMC7237067

[196] Wang S , Zhang Q , Hui H , Agrawal K , Karris MAY , Rana TM . An atlas of immune cell exhaustion in HIV‐infected individuals revealed by single‐cell transcriptomics. Emerg Microbes Infect. 2020;9:2333‐2347. 32954948 10.1080/22221751.2020.1826361 PMC7646563

[197] Nowakowski TJ , Pollen AA , Di Lullo E , Sandoval‐Espinosa C , Bershteyn M , Kriegstein AR . Expression analysis highlights axl as a candidate zika virus entry receptor in neural stem cells. Cell Stem Cell. 2016;18:591‐596. 27038591 10.1016/j.stem.2016.03.012 PMC4860115

[198] Onorati M , Li Z , Liu F , et al. Zika virus disrupts phospho‐tbk1 localization and mitosis in human neuroepithelial stem cells and radial glia. Cell Rep. 2016;16:2576‐2592. 27568284 10.1016/j.celrep.2016.08.038 PMC5135012

[199] Zanini F , Pu SY , Bekerman E , Einav S , Quake SR . Single‐cell transcriptional dynamics of flavivirus infection. Elife. 2018;7:e32942. 29451494 10.7554/eLife.32942 PMC5826272

[200] Cao Y , Su B , Guo X , et al. Potent neutralizing antibodies against SARS‐CoV‐2 identified by high‐throughput single‐cell sequencing of convalescent patients' b cells. Cell. 2020;182:73‐84.e16. 32425270 10.1016/j.cell.2020.05.025 PMC7231725

[201] Grant RA , Morales‐Nebreda L , Markov NS , et al. Circuits between infected macrophages and t cells in SARS‐CoV‐2 pneumonia. Nature. 2021;590:635‐641. 33429418 10.1038/s41586-020-03148-w PMC7987233

[202] Liao M , Liu Y , Yuan J , et al. Single‐cell landscape of bronchoalveolar immune cells in patients with COVID‐19. Nat Med. 2020;26:842‐844. 32398875 10.1038/s41591-020-0901-9

[203] Melms JC , Biermann J , Huang H , et al. A molecular single‐cell lung atlas of lethal COVID‐19. Nature. 2021;595:114‐119. 33915568 10.1038/s41586-021-03569-1 PMC8814825

[204] Ren X , Wen W , Fan X , et al. COVID‐19 immune features revealed by a large‐scale single‐cell transcriptome atlas. Cell. 2021;184:1895‐1913.e1819. 33657410 10.1016/j.cell.2021.01.053 PMC7857060

[205] Sinha S , Rosin NL , Arora R , et al. Dexamethasone modulates immature neutrophils and interferon programming in severe COVID‐19. Nat Med. 2022;28:201‐211. 34782790 10.1038/s41591-021-01576-3 PMC8799469

[206] Sungnak W , Huang N , Bécavin C , et al. SARS‐CoV‐2 entry factors are highly expressed in nasal epithelial cells together with innate immune genes. Nat Med. 2020;26:681‐687. 32327758 10.1038/s41591-020-0868-6 PMC8637938

[207] Tang X , Uhl S , Zhang T , et al. SARS‐CoV‐2 infection induces beta cell transdifferentiation. Cell Metab. 2021;33:1577‐1591.e1577. 34081913 10.1016/j.cmet.2021.05.015 PMC8133495

[208] Cristinelli S , Ciuffi A . The use of single‐cell RNA‐seq to understand virus–host interactions. Curr Opin Virol. 2018;29:39‐50. 29558678 10.1016/j.coviro.2018.03.001

[209] Justet A , Zhao AY , Kaminski N . From COVID to fibrosis: lessons from single‐cell analyses of the human lung. Hum Genomics. 2022;16:20. 35698166 10.1186/s40246-022-00393-0 PMC9189802

[210] Ratnasiri K , Wilk AJ , Lee MJ , Khatri P , Blish CA . Single‐cell RNA‐seq methods to interrogate virus–host interactions. Semin Immunopathol. 2022;45:71‐89. 36414692 10.1007/s00281-022-00972-2 PMC9684776

[211] Song H , Seddighzadeh B , Cooperberg MR , Huang FW . Expression of ace2, the SARS‐CoV‐2 receptor, and tmprss2 in prostate epithelial cells. Eur Urol. 2020;78:296‐298. 32418620 10.1016/j.eururo.2020.04.065 PMC7200365

[212] Zhao Y , Zhao Z , Wang Y , Zhou Y , Ma Y , Zuo W . Single‐cell RNA expression profiling of ace2, the receptor of SARS‐CoV‐2. Am J Respir Crit Care Med. 2020;202:756‐759. 32663409 10.1164/rccm.202001-0179LE PMC7462411

[213] Zhou L , Niu Z , Jiang X , et al. SARS‐CoV‐2 targets by the pscrna profiling of ace2, tmprss2 and furin proteases. iScience. 2020;23:101744. 33134888 10.1016/j.isci.2020.101744 PMC7591870

[214] Aguilar C , Alves da Silva M , Saraiva M , Neyazi M , Olsson IAS , Bartfeld S . Organoids as host models for infection biology—a review of methods. Exp Mol Med. 2021;53:1471‐1482. 34663936 10.1038/s12276-021-00629-4 PMC8521091

[215] Dutta D , Clevers H . Organoid culture systems to study host–pathogen interactions. Curr Opin Immunol. 2017;48:15‐22. 28756233 10.1016/j.coi.2017.07.012 PMC7126332

[216] Hicks SC , Townes FW , Teng M , Irizarry RA . Missing data and technical variability in single‐cell RNA‐sequencing experiments. Biostatistics (Oxford, England). 2018;19:562‐578. 29121214 10.1093/biostatistics/kxx053 PMC6215955

[217] Mylka V , Matetovici I , Poovathingal S , et al. Comparative analysis of antibody‐ and lipid‐based multiplexing methods for single‐cell RNA‐seq. Genome Biol. 2022;23:55. 35172874 10.1186/s13059-022-02628-8 PMC8851857

[218] Shin D , Lee W , Lee JH , Bang D . Multiplexed single‐cell RNA‐seq via transient barcoding for simultaneous expression profiling of various drug perturbations. Sci Adv. 2019;5:eaav2249. 31106268 10.1126/sciadv.aav2249 PMC6520024

[219] Byeon SK , Madugundu AK , Garapati K , et al. Development of a multiomics model for identification of predictive biomarkers for COVID‐19 severity: a retrospective cohort study. Lancet Digit Health. 2022;4:e632‐e645. 35835712 10.1016/S2589-7500(22)00112-1 PMC9273185

[220] Du M , Cai G , Chen F , Christiani DC , Zhang Z , Wang M . Multiomics evaluation of gastrointestinal and other clinical characteristics of COVID‐19. Gastroenterology. 2020;158:2298‐2301.e2297. 32234303 10.1053/j.gastro.2020.03.045 PMC7270476

[221] Wilk AJ , Lee MJ , Wei B , et al. Multi‐omic profiling reveals widespread dysregulation of innate immunity and hematopoiesis in COVID‐19. J Exp Med. 2021;218:e20210582. 34128959 10.1084/jem.20210582 PMC8210586

[222] Bernardes JP , Mishra N , Tran F , et al. Longitudinal multi‐omics analyses identify responses of megakaryocytes, erythroid cells, and plasmablasts as hallmarks of severe COVID‐19. Immunity. 2020;53:1296‐1314. 33296687 10.1016/j.immuni.2020.11.017 PMC7689306

[223] Li CX , Gao J , Zhang Z , et al. Multiomics integration‐based molecular characterizations of COVID‐19. Brief Bioinform. 2022;23:bbab485. 34864875 10.1093/bib/bbab485 PMC8769889

[224] Tan HW , Xu YM , Lau ATY . Human bronchial‐pulmonary proteomics in coronavirus disease 2019 (COVID‐19) pandemic: applications and implications. Expert Rev Proteomics. 2021;18:925‐938. 34812694 10.1080/14789450.2021.2010549

[225] Tang Z , Fan W , Li Q , et al. Mvip: multi‐omics portal of viral infection. Nucleic Acids Res. 2022;50:D817‐D827. 34718748 10.1093/nar/gkab958 PMC8689837

[226] Tian Y , Carpp LN , Miller HER , Zager M , Newell EW , Gottardo R . Single‐cell immunology of SARS‐CoV‐2 infection. Nat Biotechnol. 2022;40:30‐41. 34931002 10.1038/s41587-021-01131-y PMC9414121

[227] Unterman A , Sumida TS , Nouri N , et al. Single‐cell multi‐omics reveals dyssynchrony of the innate and adaptive immune system in progressive COVID‐19. Nat Commun. 2022;13:440. 35064122 10.1038/s41467-021-27716-4 PMC8782894

