# Supplementary material for: Development trends of human organoid‐based COVID‐19 research based on bibliometric analysis
Source: Cell Prolif. 2023 May 22;56(12):e13496. doi: 10.1111/cpr.13496 (PMC10693193; doi:10.1111/cpr.13496)
Supplement: Supplementary file 1 — Appendix S1: Supporting information. [file CPR-56-e13496-s001.docx]

**Supplementary data**

**Development trends of human organoid-based COVID-19 research based on bibliometric analysis**

Minghui Li^1, 2 *^, Yuhan Yuan^1^, Ting Zou^2^, Zongkun Hou^3^, Bochu Wang^1, *^

1. Key Laboratory of Biorheological Science and Technology, Ministry of Education, College of Bioengineering, Chongqing University, Chongqing 400030, China
2. Southwest Hospital/Southwest Eye Hospital, Third Military Medical University (Army Medical University), Chongqing 400038, China
3. School of Basic Medical Sciences/School of Biology and Engineering (School of Modern Industry for Health and Medicine), Guizhou Medical University, Guiyang 550025, China

*Corresponding author:

[mhli1988@outlook.com](mailto:shilei_hao@cqu.edu.cn) (M.H); wangbc2000@cqu.edu.cn (B.W.)


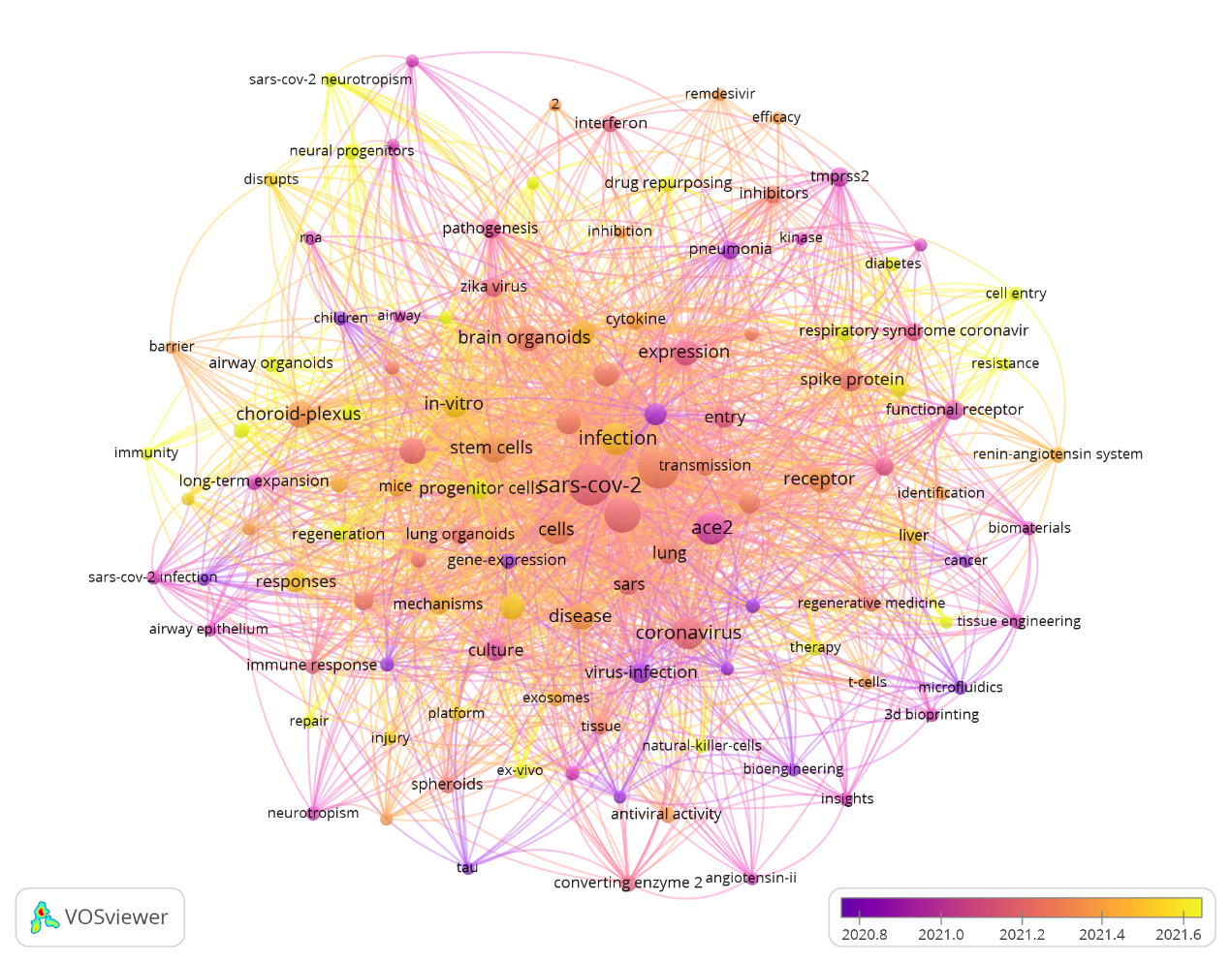


**Fig. S1. Keyword co-occurrence (at least three times) overlay visualization of the research of organoids for SARS-CoV-2 infection.** Node size indicates the occurrence frequency; Node color represents the keyword co-occurrence based on their average publication year, purple means that keywords occurred earlier and marked with yellow stands that keywords are vigorous recently; Cluster resolution = 1.00.

**Table S1. The top 10 contributing institutions regarding organoid-based COVID-19 research.**

| **Rank** | **Institutions** | **Publications** | **Average Citations** | **H-index** |
| --- | --- | --- | --- | --- |
| 1 | UNIVERSITY OF CALIFORNIA SYSTEM | 21 | 43.9 | 12 |
| 2 | CHINESE ACADEMY OF SCIENCES | 15 | 19.53 | 6 |
| 3 | UNIVERSITY OF CALIFORNIA SAN DIEGO | 12 | 37.67 | 9 |
| 4 | CORNELL UNIVERSITY | 11 | 54 | 5 |
| 5 | ERASMUS MC | 10 | 114.9 | 8 |
| 6 | ERASMUS UNIVERSITY ROTTERDAM | 10 | 114.9 | 8 |
| 7 | HARVARD UNIVERSITY | 10 | 22.2 | 4 |
| 8 | HARVARD MEDICAL SCHOO | 9 | 24.67 | 4 |
| 9 | KAROLINSKA INSTITUTET | 9 | 165.22 | 5 |
| 10 | UNIVERSITY OF HONG KONG | 9 | 39.67 | 5 |
